# SLC27A5 promotes sorafenib-induced ferroptosis in hepatocellular carcinoma by downregulating glutathione reductase

**DOI:** 10.1038/s41419-023-05558-w

**Published:** 2023-01-12

**Authors:** Feng-li Xu, Xiao-hong Wu, Chang Chen, Kai Wang, Lu-yi Huang, Jie Xia, Yi Liu, Xue-feng Shan, Ni Tang

**Affiliations:** 1 Key Laboratory of Molecular Biology for Infectious Diseases (Ministry of Education), Institute for Viral Hepatitis, Department of Infectious Diseases, The Second Affiliated Hospital, Chongqing Medical University, Chongqing, China; 2 Institute of Life Sciences, Chongqing Medical University, Chongqing, China; 3 Department of Pharmacy, The First Affiliated Hospital, Chongqing Medical University, Chongqing, China

**Keywords:** Cancer therapeutic resistance, Oncogenes

## Abstract

Sorafenib, a first-line drug for advanced hepatocellular carcinoma (HCC), shows a favorable anti-tumor effect while resistance is a barrier impeding patients from benefiting from it. Thus, more efforts are needed to lift this restriction. Herein, we first find that solute carrier family 27 member 5 (SLC27A5/FATP5), an enzyme involved in the metabolism of fatty acid and bile acid, is downregulated in sorafenib-resistant HCC. SLC27A5 deficiency facilitates the resistance towards sorafenib in HCC cells, which is mediated by suppressing ferroptosis. Further mechanism studies reveal that the loss of SLC27A5 enhances the glutathione reductase (GSR) expression in a nuclear factor erythroid 2-related factor 2 (NRF2)-dependent manner, which maintains glutathione (GSH) homeostasis and renders insensitive to sorafenib-induced ferroptosis. Notably, SLC27A5 negatively correlates with GSR, and genetic or pharmacological inhibition of GSR strengthens the efficacy of sorafenib through GSH depletion and the accumulation of lipid peroxide products in SLC27A5-knockout and sorafenib-resistant HCC cells. Based on our results, the combination of sorafenib and carmustine (BCNU), a selective inhibitor of GSR, remarkably hamper tumor growth by enhancing ferroptotic cell death in vivo. In conclusion, we describe that SLC27A5 serves as a suppressor in sorafenib resistance and promotes sorafenib-triggered ferroptosis via restraining the NRF2/GSR pathway in HCC, providing a potential therapeutic strategy for overcoming sorafenib resistance.

## Introduction

Liver cancer, of which hepatocellular carcinoma (HCC) is the primary subtype, is the fourth most lethal tumor worldwide [1]. The prevalence of liver cancer has shown an increase and more than a million people will be challenged with it by 2025 [2, 3]. Risk factors contributing to HCC include hepatitis B and C virus infection, alcohol abuse, and non-alcoholic steatohepatitis (NASH) which is an early warning of HCC to patients with metabolic diseases [3]. Potentially curative treatment is the optimal candidate therapy that consists of tumor resection, location ablation, and liver transplantation for early-stage patients. As for advanced HCC, systemic therapies are considered to be the major solution [4]. Sorafenib, a multi-target kinase inhibitor, is the FDA-approved first-line systemic therapy for the advanced HCC and prolongs the median overall survival to 6.5 months [5]. However, in addition to some patients who showed a low response rate named primary resistance, the benefits of sorafenib are limited in those who are sensitive at the beginning of treatment on account of acquired resistance within 6 months [6, 7]. Previous work showed that there is an inherent association between resistance and genetic alterations. Meanwhile, several genetic changes also contribute to the development of HCC [6]. Thus, uncovering the resistance target and combination drugs are the essential entry point to breaking the dilemma of sorafenib resistance [8].

Ferroptosis, an emerging focus firstly described by Dixon and his colleagues, is iron-dependent regulated cell death that is driven by the lethal accumulation of lipid peroxides [9, 10]. Reduced numbers of mitochondrial cristae and shrunken mitochondria are unique morphology characteristics that distinguish ferroptosis from other cell death forms [11]. Mechanistically lipid peroxides undertake the mission of ferroptosis execution, which is the confrontation between the generation of oxidized phospholipid and the ferroptosis suppressing system mainly based on glutathione (GSH)-dependent and GSH-independent manners [12–15]. Growing evidence demonstrated that sorafenib is an inducer of ferroptosis independently of RAF kinase inhibitory effect [16–18]. It is reported that the cystine-glutamate antiporter-xCT is suppressed on exposure to sorafenib, leading to the accumulation of ROS and GSH depletion [17]. As such, targeting ferroptosis vulnerability may be a prospective therapeutic approach for sorafenib resistance.

The solute carrier family 27 (SLC27), comprises six members and expresses in various tissues with active fatty acid and lipid metabolism [19, 20]. The solute carrier family 27 member 5 (SLC27A5) is distinctive among family members in that is exclusively expressed in the basement membrane of the liver and exhibits bile acid-CoA ligase activity [21, 22]. As the liver is the central organ of fatty acid and bile metabolism, SLC27A5 plays a prominent role in many pathological conditions in the liver, including NASH, cirrhosis, and cholestasis [23–26]. Our previous work has shown that SLC27A5, inactivated by promoter hypermethylation, is a tumor suppressor gene in HCC [27]. Silencing of SLC27A5 promotes tumor progression by antioxidant and oncogenic signaling pathways and is associated with poor prognosis [27–29].

Herein, our work aimed at dissecting the role and underlying mechanism of SLC27A5 deficiency in resisting sorafenib. Further decreased SLC27A5 conferred resistance to sorafenib-induced ferroptosis through activating glutathione reductase (GSR). Our findings suggested that a combination regime of sorafenib and GSR inhibitor- carmustine (BCNU) may prove to be a novel strategy for the resistance of HCC.

## Results

### SLC27A5 is downregulated in sorafenib-resistant hepatoma cells

**Fig. 1:**
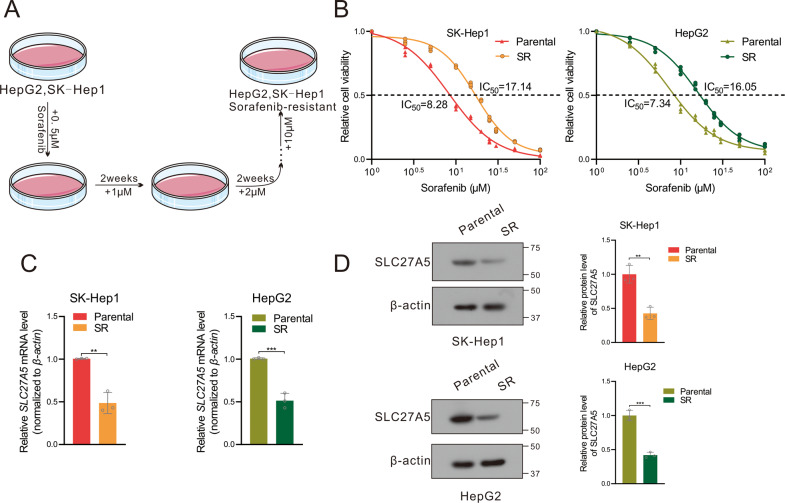
SLC27A5 expression is downregulated in the sorafenib-resistant hepatocellular carcinoma cells. **A** Schematic diagram of the construction of the sorafenib-resistant cells. **B** The IC_50_ values of sorafenib-sensitive and sorafenib-resistant SK-Hep1 and HepG2 cells that were incubated with sorafenib in a concentration gradient manner. **C**, **D** The mRNA (**C**) and protein (**D**) expression of SLC27A5 in sorafenib-sensitive and sorafenib-resistant HCC cells. SR sorafenib resistant. All data are presented as mean ± SD (*n* = 3). Statistical significance was calculated using two-tailed unpaired Student’s *t-*test. *p < 0.05, **p < 0.01, ***p < 0.001.

To figure the role of SLC27A5 in sorafenib resistance, we performed in-depth data mining using the Gene Expression Omnibus (GEO) database, in which the mRNA level of *SLC27A5* was downregulated in sorafenib-resistant Huh7 (Fig. S1A) and even in HepG2 that incubated with sorafenib (Fig. S1B). As an essential tool to dig the potential molecular mechanisms of sorafenib resistance, sorafenib-resistant HCC lines were constructed by being exposed to sorafenib treatment for 2–3 days, and resistance was induced by adding step-wise elevating the sorafenib concentration into the medium during repeated passages [30] (Fig. 1A). The acquired resistance of these cells was determined by comparison with their parental counterparts. The half-maximal inhibitory concentrations (IC_50_) values of sorafenib-resistant SK-Hep1 (SK-SR) and sorafenib-resistant HepG2 (HepG2-SR) were 17.14 μM and 16.05 μM respectively which were notably higher than that of parental cells (Fig. 1B). Importantly, we found that the level of SLC27A5 was dramatically reduced in sorafenib-resistant HCC cells at the mRNA levels as well as protein levels (Fig. 1C, D). Altogether, the data presented above indicate that SLC27A5 is downregulated in sorafenib-resistant HCC cells.

### Loss of SLC27A5 enhances the resistance of HCC cells to sorafenib

**Fig. 2:**
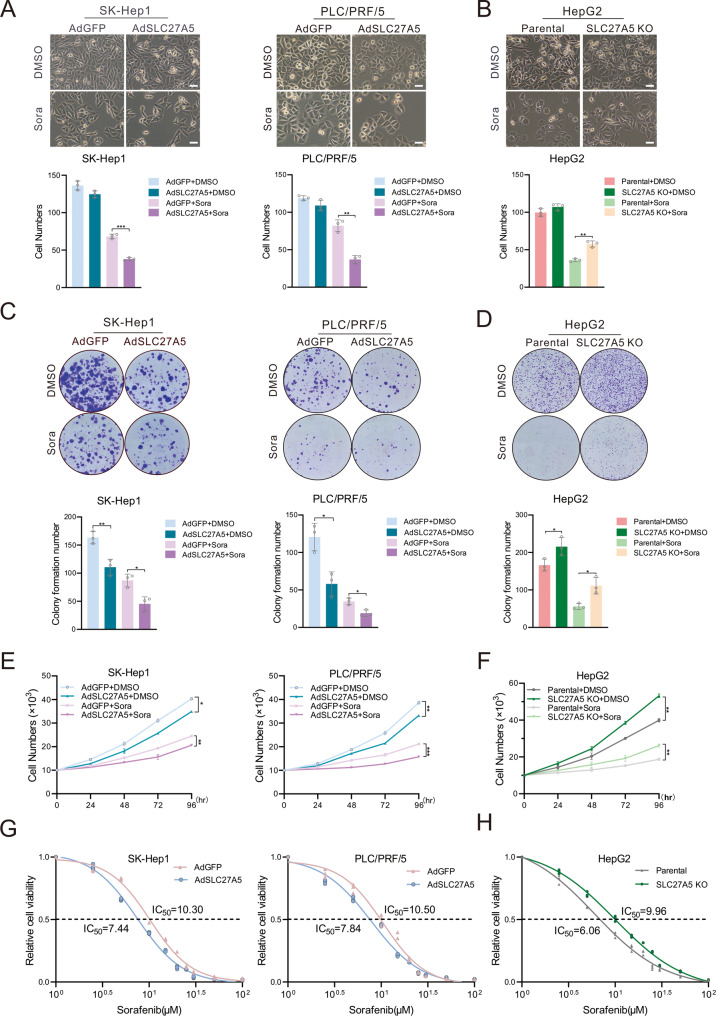
Knockout of SLC27A5 enhances sorafenib resistance in HCC cells. **A**, **B** Representative morphological images (top) and quantification (bottom) of ectopic expression SLC27A5 (**A**) and SLC27A5-KO (**B**) cells treated with DMSO or sorafenib (10 μM) for 24 h. **C**, **D** Colony formation assay (top) of SLC27A5 overexpression (**C**) and SLC27A5-KO (**D**) HCC cells for 14 days as well as quantification of clusters (bottom). Scale bar: 10 μm. **E**, **F** Cell growth curves of SLC27A5-overexpression (**E**) and SLC27A5-KO (**F**) cells for 4 days. **G**, **H** The IC_50_ values of SLC27A5-overexpression (**G**) and SLC27A5-KO (**H**) cells were measured by the CCK-8 assays with the indicated concentration of sorafenib for 24 h. DMSO dimethyl sulfoxide, Sora sorafenib. Values represent the mean ± SD (*n* = 3). Statistical significance was calculated using two-tailed unpaired Student’s *t-*test. *p < 0.05, **p < 0.01 ***p < 0.001.

Since the low expression of SLC27A5 in sorafenib-resistant hepatoma cells, we sought to focus on analyzing whether SLC27A5 is functionally involved in sorafenib resistance. Thus next, SK-Hep1 and PLC/PRF/5 were infected with recombinant adenovirus expressing SLC27A5 (AdSLC27A5), while AdGFP was used as control [27] (Fig. S2A, B). Knockout of SLC27A5 in HepG2 cells was achieved using the CRISPR/Cas9 system [27] (Fig. S2C). The ectopic expression of SLC27A5 reduced cell survival after sorafenib treatment, whereas that of SLC27A5-KO was enhanced (Fig. 2A, B). Furthermore, overexpression of SLC27A5 moderately inhibited cell growth of HCC cells under the treatment of sorafenib as shown in colony formation and growth curves. Reverse effects were observed in SLC27A5-KO cells (Fig. 2C–F). As expected, the values of IC_50_ showed that overexpression of SLC27A5 sensitizes HCC cells to sorafenib (Fig. 2G), and knockout of SLC27A5 attenuates the inhibitory effect of sorafenib (Fig. 2H). Collectively, these results illustrate that reduced expression of SLC27A5 in HCC cells increases tolerance to sorafenib.

### SLC27A5-dependent sorafenib resistance is mediated by ferroptosis in HCC

**Fig. 3:**
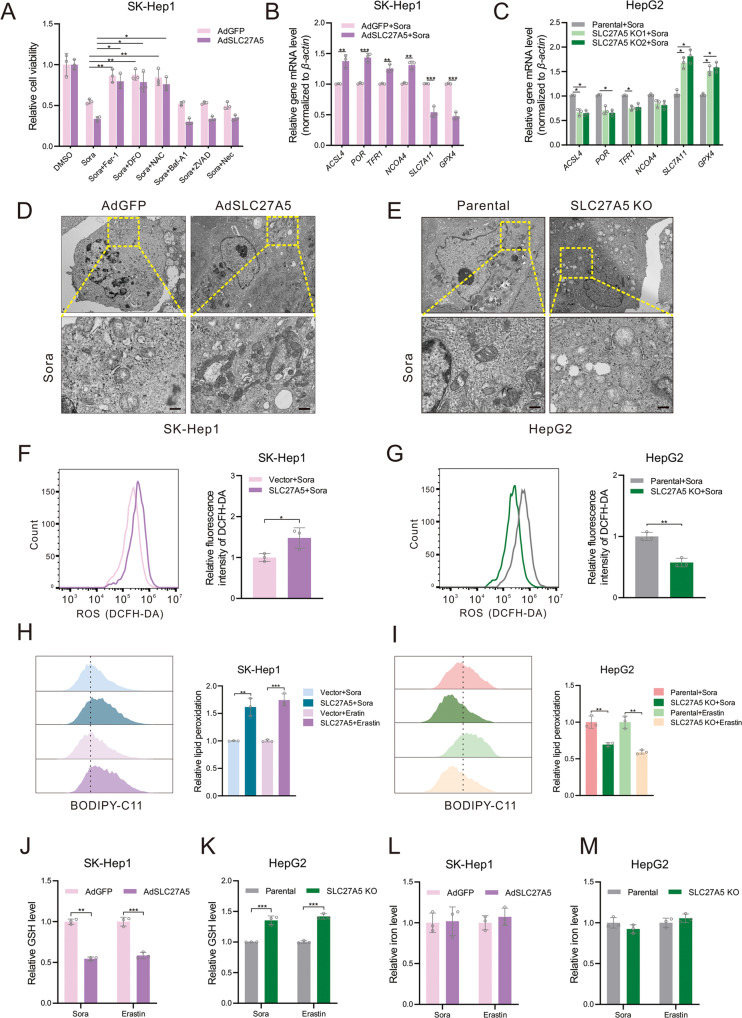
SLC27A5-deficiency-induced sorafenib resistance is mediated by ferroptosis in HCC. **A** SLC27A5-overexpression cells were cultured with sorafenib (10 μM) alone or co-treatment with sorafenib and anyone cell death inhibitor (Fer-1, 1 μM; DFO, 100 μM; NAC, 10 mM, Baf-A1, 0.1 μM; ZVAD-FMK, 10 μM; Necrosulfonamide, 0.5 μM) for 24 h. **B**, **C** The relative mRNA level of biomarker genes related to ferroptosis by RT-qPCR. **D**, **E** The mitochondria of SK-Hep1 with overexpressed SLC27A5 (**D**) and SLC27A5-KO HepG2 (**E**) were analyzed by electron micrographs. Scale bar: 1 μm. **F**, **G** Flow cytometry was performed to analyze the relative ROS level using DCFH-DA probe in SK-Hep1 cells transfected with pSEB-3Flag-SLC27A5 (**F**) or in SLC27A5-KO HepG2 (**G**) cells. **H**, **I** The level of lipid peroxidation was assayed by BODIPY-C11 in SK-Hep1 cells transfected with pSEB-3Flag-SLC27A5 (**H**) or in SLC27A5-KO HepG2 (**I**) cells. **J**–**M** Relative level of GSH and Fe^2+^ in SLC27A5-overexpressing (**J**, **L**) and SLC27A5-KO (**K**, **M**) HCC cells. Fer-1 ferrostatin-1, DFO deferoxamine, NAC n-acetyl-l-cysteine, Baf-A1 bafilomycin A1, Nec necrosulfonamide, ROS reactive oxygen species, GSH glutathione, MDA malondialdehyde. Quantitative data are represented as the mean ± SD (*n* = 3). Statistical significance was calculated using two-tailed unpaired Student’s *t* test and one-way ANOVA test. *p < 0.05, **p < 0.01, ***p < 0.001.

According to available reports, the types of cell death induced by sorafenib include apoptosis [31], ferroptosis [17], and autophagy [32], which occupy important positions in sorafenib resistance [8]. To investigate the underlying resistance mechanism of SLC27A5, we got SLC27A5-overexpression HCC cells to incubate with widely recognized cell death inhibitors and measured the inhibitory effects on cell viability. As shown in the result, it was the ferroptosis inhibitors (ferrostatin-1, Deferoxamine, N-Acetyl-L-cysteine) that restored cell viability but not other cell death inhibitors, including Bafilomycin A1 (autophagy inhibitor), Z-VAD-FMK (apoptosis inhibitor), Necrosulfonamide (necroptosis inhibitor) (Figs. 3A and S3A). These findings prompted us to observe the hallmarks of ferroptosis in these HCC cell lines. The imbalance of lipid peroxidation and antioxidant systems is a critical cause of ferroptosis [33]. Therefore, following previous studies, we observed variations in a list of genes that promote and inhibit ferroptosis [9, 12, 34–37]. Enforced expression of SLC27A5 contributed to the upregulation of genes promoting lipid peroxidation and dysregulation of iron homeostasis (Figs. 3B and S3B). Meanwhile, deficiency of SLC27A5 resulted in resistance to sorafenib-induced ferroptosis (Fig. 3C). Moreover, mitochondrial morphology of SLC27A5-overexpressing HCC cells manifested as decreased even vanished mitochondria cristae, and increased mitochondrial membrane density compared to the control group (Fig. 3D). Conversely, knockout of SLC27A5 kept HCC cells with relatively normal mitochondrial morphology after treatment of sorafenib (Fig. 3E). Afterward, the levels of reactive oxygen species (ROS) and lipid peroxidation were modestly elevated with forced expression of SLC27A5, whereas the SLC27A5-KO alleviated the oxidative stress (Figs. 3F–I and S3C–F). Consistent with the above results, the relative content of reduced glutathione (GSH) was remarkably reduced in SLC27A5-overexpressing cells (Figs. 3J and S3G) and elevated with the silence of SLC27A5 (Fig. 3K). In addition, ferrous iron (Fe^2+^) is a significant participant in the Fenton reaction which promoted ROS generation and induced ferroptosis [38]. Results showed that SLC27A5 interference did not affect the Fe^2+^ levels under the treatment of sorafenib or erastin (Figs. 3L, M and S3H). Above all, our findings suggest that ferroptosis caused by GSH depletion-mediated lipid peroxidation contributes to SLC27A5-deficiency-induced sorafenib resistance in HCC.

### SLC27A5 overexpression overcomes the resistance of HCC-SR cells towards sorafenib by inducing ferroptosis

**Fig. 4:**
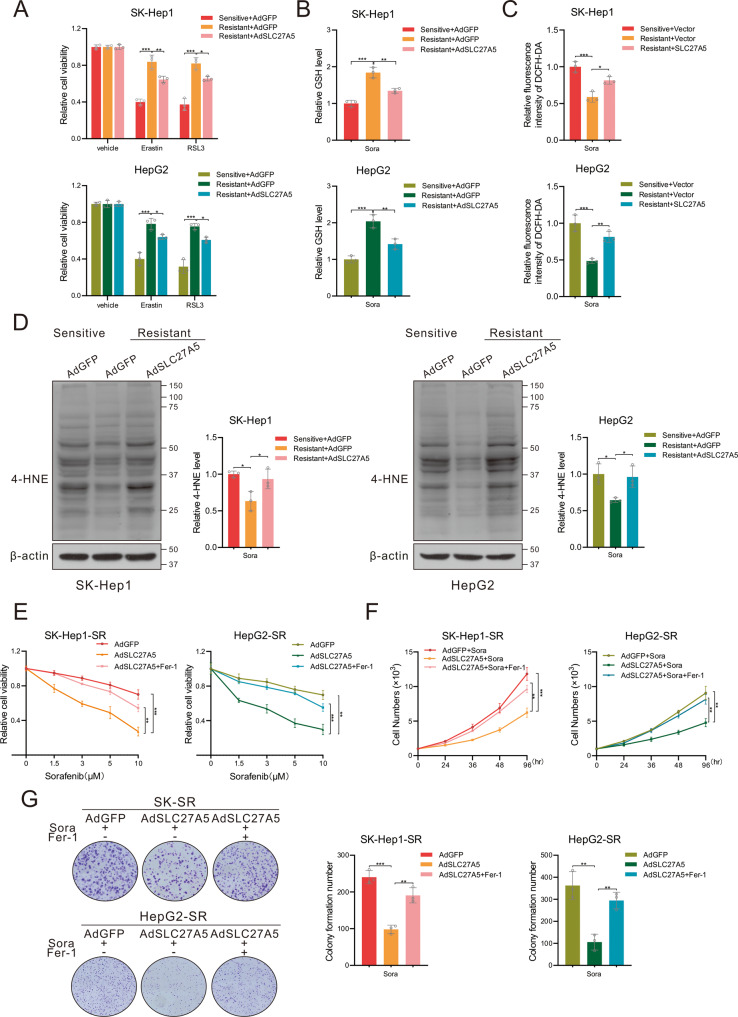
Enforced SLC27A5 expression sensitizes HCC-SR cells to sorafenib. **A** Relative cell viability of SLC27A5-overexpression sorafenib-resistant HCC cells treated with Erastin (10 μM) or RSL3 (0.5 μM) for 24 h. **B** Measurement of intracellular GSH levels in SLC27A5-overexpression sorafenib-resistant HCCs. **C** Relative level of intracellular ROS in sorafenib-resistant HCC cells transfected with pSEB-3Flag-SLC27A5. **D** 4-HNE-induced protein modification was detected by immunoblotting. **E** SLC27A5-overexpression sorafenib-resistant HCC cells were treated with various concentrations of sorafenib and 1 μM Fer-1 for 48 h. The viability of cells was determined by the CCK-8 assay. **F**, **G** Growth curves (**F**) and clone formation (**G**) of SLC27A5-overexpression sorafenib-resistant HCC cells incubated with sorafenib (10 μM) alone or sorafenib plus Fer-1 (1 μM). 4-HNE: 4-hydroxy-2-nonenal. Values represent the mean ± SD (*n* = 3). Statistical significance was calculated using the one-way ANOVA test. *p < 0.05, **p < 0.01 ***p < 0.001.

Sorafenib induces the generation of ROS in cancer cells, which interacts with biological macromolecules and disturbs cellular metabolic homeostasis [39, 40]. However, reduced ROS levels and resistance to ferroptosis-inducing drugs were observed in sorafenib-resistant cells compared to their sensitive counterparts [41, 42]. To assess the role of ferroptosis in sorafenib-resistant cells, HCC-SR cells were challenged with two ferroptosis inducers, including erastin and RSL-3. As expected, the HCC-SR cells were more insensitive to ferroptosis induction in contrast to the sensitive cells but became vulnerable to erastin and RSL-3 upon SLC27A5 re-expression (Figs. 4A and S4A, B). Likewise, decreased levels of GSH accompanied by increased accumulation of ROS and intracellular 4-hydroxy-2-nonenal (4-HNE) were found in SLC7A5-overexpressing resistant cells (Figs. 4B–D and S4C–F) under the treatment of sorafenib, another ferroptosis inducer. And then, we measured the proliferation in resistant HCC cells with overexpression of SLC27A5. Indeed, overexpression of SLC27A5 conferred the sensitivity to sorafenib treatment in resistant cells, as shown by relative cell viability, growth curves, and colony formation (Fig. 4E–G). Furthermore, these phenomena raised by SLC27A5 overexpression were recovered by co-treatment with ferrostatin-1(Fer-1), a ferroptosis inhibitor (Fig. 4E–G). These data reinforce that SLC27A5 may promote sorafenib-induced ferroptosis.

### SLC27A5 negatively regulates NRF2/GSR signaling pathway in HCC

**Fig. 5:**
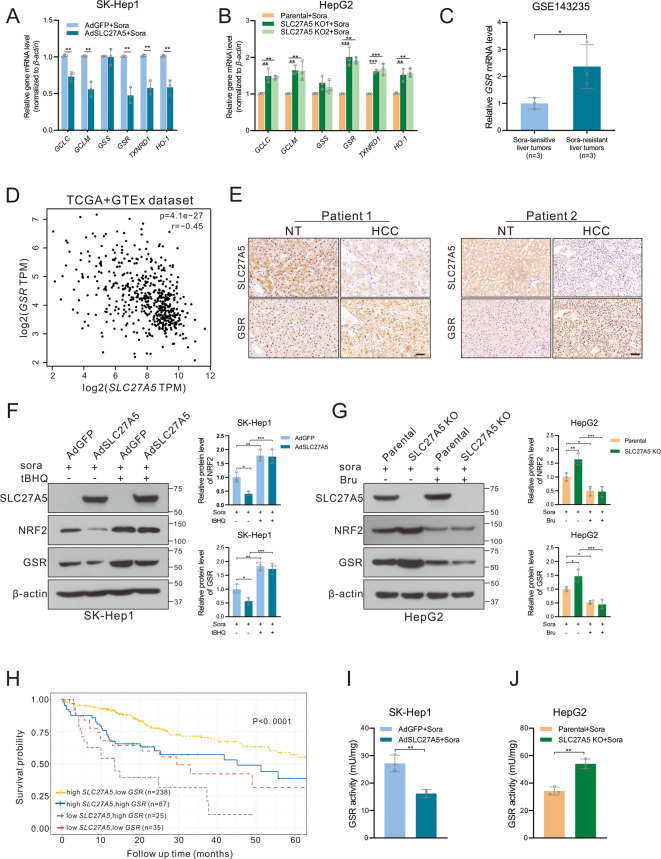
SLC27A5 depletion activated NRF2/GSR axis in HCC. **A**, **B** Representative mRNA levels of NRF2 downstream gene expression in SLC27A5-overexpression (**A**) and SLC27A5-KO (**B**) cells under treatment of sorafenib. **C** Relative mRNA levels of *GSR* in sorafenib-sensitive (*n* = 3) and sorafenib-resistant (*n* = 3) human liver tumors. **D** Correlation analysis of the mRNA levels of *SLC27A5* and *GSR* in the liver by Spearman. **E** Histochemical staining for SLC27A5 and GSR in HCC tissues and adjacent non-cancerous tissues. Scale bar: 50 μm. **F** Relative protein expression of NRF2 and GSR were detected by western blotting in SLC27A5-overexpression SK-Hep1 cultured with sorafenib (10 μM for 24 h) alone or co-treatment with tBHQ (100 μM for 3 h). **G** Relative protein expression of NRF2 and GSR were detected by western blotting in SLC27A5-KO HepG2 cultured with sorafenib alone or co-treatment with brusatol (40 nM for 24 h). **H** Kaplan–Meier survival curve analysis based on the expression of *SLC27A5* and *GSR* in the liver tumor. **I**, **J** The activity of GSR was determined by the GSR activity kit in SLC27A5-overexpression SK-Hep1 (**I**) and SLC27A5-KO HepG2 (**J**). TPM transcripts per million, tBHQ tertiary butylhydroquinone, Bru brusatol. Data shown are mean ± SD (*n* = 3). Statistical significance was calculated using two-tailed unpaired Student’s *t*-test and one-way ANOVA test. *p < 0.05, **p < 0.01 ***p < 0.001.

Our previous study reported that SLC27A5 deficiency accelerated the dissociation of NRF2 from KEAP1, which in turn enhanced antioxidative capacity [27]. NRF2, as a crucial transcription factor, activates indicated genes that widely participated in the ferroptosis-related essential process [43] and confers resistance to cancer therapy as well [44]. Based on these reports, we asked whether loss of SLC27A5 promoted sorafenib resistance by activating NRF2 and downstream antioxidant genes. Our hypothesis was proven by the real-time quantitative reverse-transcription PCR (RT-qPCR) (Fig. 5A, B and S5A) and we noticed one of the top up-regulated genes after the knockout of SLC27A5. GSR has been reported to be an indicator of malignancy in hepatocellular carcinoma [45] and mediates glioblastoma multiforme resistance [46]. The bioinformatics analysis demonstrated that *GSR* was upregulated in sorafenib-resistant human liver cancer tissues and high levels of *GSR* were related to poor prognosis (Figs. 5C and S5B, C). Then, data demonstrated that *GSR* is negatively correlated with *SLC27A5* (*r* = −0.45, *p* = 4.1e-27) and positively correlated with *NFE2L2* (*r* = 0.43, *p* = 2.6e-25) in TCGA and GTEx Dataset (Fig. 5D and Fig. S5D). The immunohistochemical (IHC) analysis of human HCC and adjacent non-tumor tissue sections confirmed the results of the database (Figs. 5E and S5E; Table. S2). Moreover, the western blot indicated that the protein levels of GSR were decreased in HCC cells with SLC27A5-overexpression as well (Fig. 5F). But tertiary butylhydroquinone (tBHQ), the activator of NRF2, blocked such effect under the treatment of sorafenib (Fig. 5F). In contrast, the knockout of SLC27A5 resulted in up-regulating GSR while brusatol inhibits the expression of NRF2 and GSR (Fig. 5G). Meanwhile, survival analysis of multi-genes was performed to reveal that the patients with both high levels of *SLC27A5* and low levels of *GSR* or *NFE2L2* were significantly associated with a better prognosis than others (Figs. 5H and S5F). Since GSR is a key reductase mediating reductive recycling of GSSG and GSH homeostasis, we assessed functionally the levels of enzyme activity. Results showed a consistent tendency with the levels of protein, the enzyme activity of GSR was significantly decreased in SLC27A5-overexpressing cells and increased as a result of the deficiency of SLC27A5 (Figs. 5I, J and S5G). These findings gave strong evidence to confirm that SLC27A5 deficiency leads to activation of the NRF2/GSR axis and GSR might play a crucial role in HCC progression and sorafenib resistance.

### Silencing GSR sensitizes HCC cells to sorafenib-induced ferroptosis

**Fig. 6:**
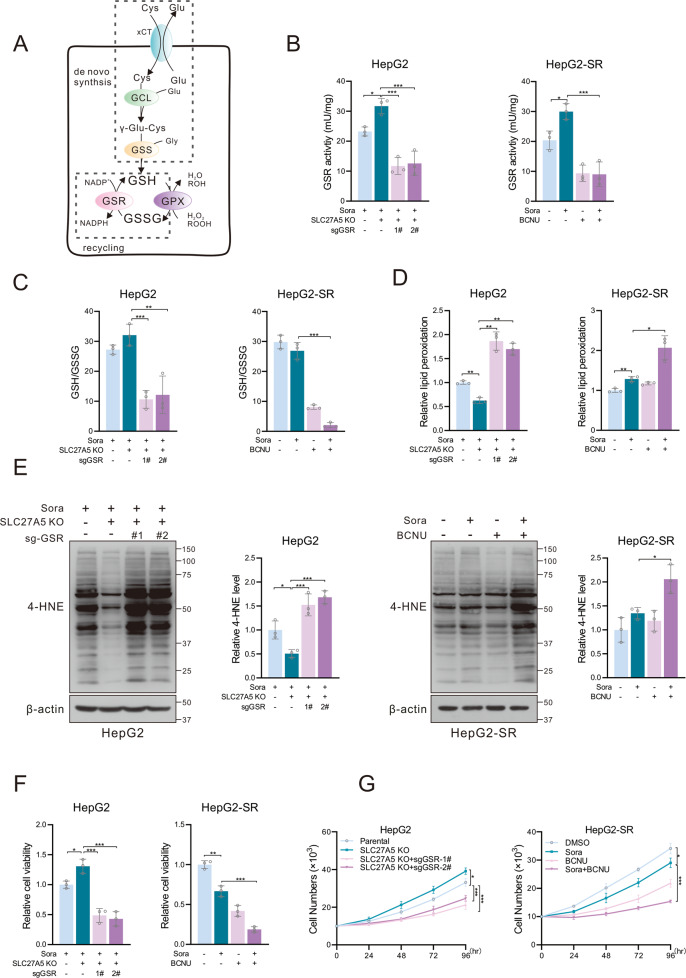
Silencing the expression of GSR sensitizes HCC cells to sorafenib. **A** Schematic diagram summarizing the de novo synthesis and recycling of GSH. **B** The enzyme activity of GSR in HepG2 transfected with sg*GSR* (left) and HepG2-SR treated with sorafenib or/and BCNU (right). **C**–**E** Relative level of GSH/GSSG ratio (**C**), lipid peroxidation (**D**), and the 4-HNE-induced protein modification (**E**) in SLC27A5-KO HepG2 transfected with sg*GSR* (left) and HepG2-SR treated with sorafenib or/and BCNU (right). **F**, **G** Relative cell viability (**F**) and cell growth curve (**G**) of SLC27A5-KO HepG2 transfected with sg*GSR* (left) and HepG2-SR treated with sorafenib or/and BCNU (right). Cys cystine, Glu glutamate, Gly glycine, BCNU carmustine. Data shown are mean ± SD (*n* = 3). Statistical significance was calculated using one-way ANOVA test. *p < 0.05, **p < 0.01 ***p < 0.001.

GSH, a thiol-containing tripeptide, is the pillar antioxidant in the antioxidative system. As the main substrate of the selenoenzyme GPX4, GSH is converted to oxidized glutathione (GSSG) for the detoxification of lipid peroxidation [12]. GSH depletion directly triggers inhibition of GPX4 activity thus cells are vulnerable to ferroptosis [47]. Levels of GSH are determined by de novo biosynthesis and by the recycling of GSSG (Fig. 6A). We assumed that cells were more rely on GSR-mediated recycling of oxidized glutathione to maintain GSH homeostasis in the condition that function deficiency in de novo synthesis mediated by sorafenib, which provided a feasible approach for overcoming the drug resistance. To further verify our conjecture, silencing *GSR* was achieved in HepG2 by using sgRNAs targeting *GSR* (sg*GSR*) (Fig. S6A). The treatment of carmustine (BCNU)- a selective inhibitor of GSR, could not alter the expression of GSR (Fig. S6B). As expected, we found that both sg*GSR* and BCNU significantly suppressed the enzyme activity as well as the GSH/GSSG ratio (Fig. 6B, C). Inhibition of GSR resulted in the depletion of reduced GSH and accumulation of lipid peroxidation in SLC27A5-KO and HepG2-SR cells (Fig. 6D, E and Fig. S6C–F). These results indicating the functional deficiency of GSR may render cells more sensitive to sorafenib-induced ferroptosis. Indeed, the resistance of SLC27A5-KO and HepG2-SR cells towards sorafenib was partially reversed due to the interference of GSH homeostasis (Fig. 6F–G). Taken together, GSR may be an attractive therapeutic target to improve the dilemma of sorafenib resistance.

### BCNU combination enhances the curative effect of sorafenib in vivo

**Fig. 7:**
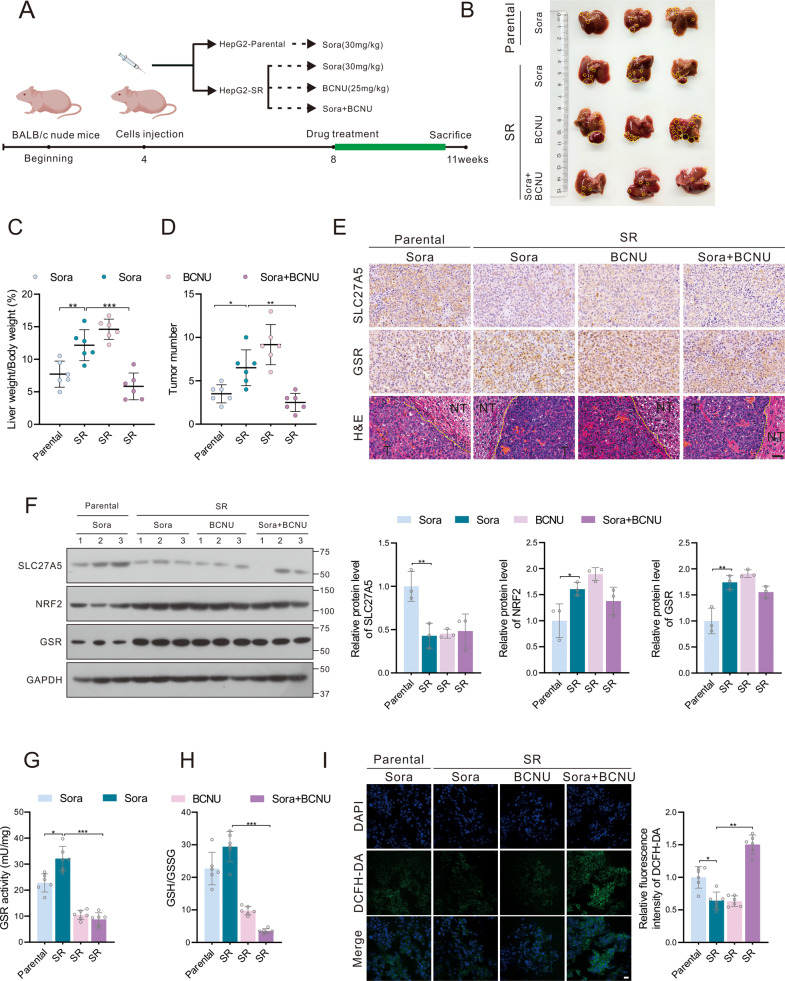
BCNU enhances the curative effect of sorafenib in vivo by inducing ferroptosis. **A** Establishment protocol for the evaluation of tumor growth and resistance of human HepG2-parental and HepG2-SR tumor xenografts in BALB/C nude mice following therapy with sorafenib and BCNU alone or both. **B** Gross images of the HepG2-derived xenografts in orthotopic implantation model. **C**, **D** Analysis of liver/body weight ratio (**C**) (*n* = 6) and tumor numbers (**D**) (*n* = 6). **E**, **F** Relative protein expression of SLC27A5 and GSR in the tumor tissues was assayed by immunohistochemistry (**E**) and immunoblotting (**F**). Scale bar: 50 μm. **G**–**I** The level of GSR activity (**G**) (*n* = 6), GSH/GSSG ratio (**H**) (*n* = 6), and ROS (**I**) (*n* = 6) were assayed in tumor tissues. Scale bar: 10 μm. Data shown are mean ± SD. Statistical significance was calculated using one-way ANOVA test. *p < 0.05, **p < 0.01, ***p < 0.001.

To further scrutinize the intrinsic association between GSR and cancer therapy, a sorafenib-resistant orthotopic xenograft model derived from HepG2 cells was established. After implantation, pharmacotherapy containing sorafenib (30 mg/kg), BCNU (25 mg/kg) alone, or both were delivered for 14 days (Fig. 7A). Compared to that of the parental group, the efficacy of sorafenib treatment was significantly attenuated in mice bearing HepG2-SR xenograft tumors (Fig. 7B–D). However, with the superposition of BCNU, sensitivity toward sorafenib of resistant hepatoma cells was restored, as indicated by restricted tumor sizes and weight. (Fig. 7B–D). Moreover, sorafenib-resistant tumors showed reduced protein levels of SLC27A5 as well as high levels of GSR (Fig. 7E, F). Following the aforementioned data in vitro, we surmised that GSH depletion mediated by the inhibition of the NRF2/GSR axis played a critical role in tumor inhibition. Consistent with our conjecture, the declining levels of GSR activity and GSH with consequent impaired the capacity of defense against oxidative stress in the combination group, allowing for the increased ROS and accumulation of MDA and 4-HNE. (Figs. 7G–I and S7A–D). These results suggested that pharmacologically blocking GSR sensitizes HCC to ferroptosis elicited by sorafenib.

## Discussion

**Fig. 8:**
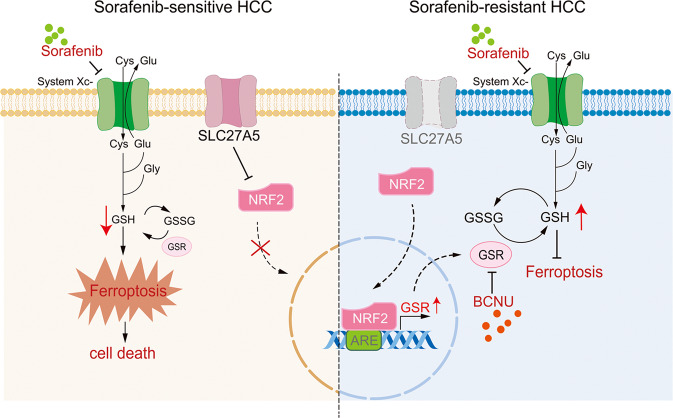
Proposed mechanistic model of sorafenib resistance caused by SLC27A5 deficiency. In sorafenib-sensitive cells, NRF2 is not activated and the restricted Cys transport and low levels of GSH result in enhanced ROS and oxidative stress. Cells are more susceptible to ferroptosis elicited by sorafenib. By contrast, SLC27A5 deficiency in sorafenib-resistant HCC cells activates the NRF2/GSR pathway to keep glutathione homeostasis and thus promotes cell survival under sorafenib treatment. GSR inhibition-BCNU significantly reduced the enzyme activity of GSR. Low GSH levels contribute to enhancing sorafenib-induced ferroptosis in HCC. ARE antioxidant response element.

In the present study, we confirmed that SLC27A5 was downregulated in sorafenib-resistant HCC cells and xenograft tumors. Faced with the menace of sorafenib resistance, SLC27A5 deficiency promotes cell survival by inhibiting ferroptosis by activating the NRF2/GSR pathway. Pharmacological or genetic inhibition of GSR significantly enhanced the execution of ferroptosis induced by sorafenib in HCC (Fig. 8). As a membrane protein that is specifically expressed in the liver, the function of SLC27A5 in normal metabolism and certain pathophysiological conditions has been well investigated. The data hinted at the vital role of SLC27A5 in sorafenib resistance in HCC therapy, whereas little is known about potential molecular mechanisms. In this work, we first try to parse the previously unknown effect of downregulated SLC27A5 in sorafenib resistance and ferroptosis execution in HCC.

Ferroptosis is driven by iron-dependent phospholipid peroxidation whose substrates are phospholipids containing polyunsaturated fatty acyl (PUFA) chains [48]. Dietary fatty acid (FA) is a vital source of arachidonic acid (AA) which is one of the most abundant PUFA species and the primary substrate of phospholipids peroxidation. PUFA including AA and adrenic acid (AdA) propagate ferroptosis induced by erastin or RSL3 [34, 49]. The uptake of lipids, a process dependent on CD36, FATPs and FA binding proteins, contributes to elevating PUFA content and ferroptosis sensitivity. Recent studies have revealed that CD36-mediated FA uptake navigates immune cells to ferroptosis [50, 51]. SLC27A5 (FATP5), another crucial FA transporter, is responsible for the uptake of LCAF and function as very long chain fatty acyl-coenzyme A synthetase [52]. SLC27A5 KO mice showed lower FA uptake and increased fatty acid synthetase expression [21, 23]. Recently, we found that SLC27A5 deficiency in HCC remodels the lipid profiles and activates the NRF2/TXNRD1 pathway [27]. As such, hepatic tumor cells were more adapted to the high levels of oxidative stress and acquired more robust proliferation [27]. Given its crucial role in lipid metabolism and HCC development, it is yet unknown whether and how SLC27A5 regulates ferroptosis through lipid metabolism. In addition, the lack of validation on clinical sorafenib-resistant HCC samples is a limitation. Future in-depth analyses should be performed to refine the role of SLC27A5 in sorafenib resistance and ferroptosis.

The participation of GSR in tumors has been previously reported. For example, the levels of GSR were markedly elevated in temozolomide (TMZ)-resistant glioma cells, indicating GSR may be a critical target in drug resistance of tumors [46]. Furthermore, GSR in human cervical cancer tissues was significantly upregulated, whereas GSR knockdown results in ROS-dependent cell death [53]. GSR depletion renders human lung cancer cells more sensitive to TXNRD1 inhibitor-Auranofin [54]. In contrast, upregulation of GSR contributed to the tolerance of colorectal cancer cells to the acidic environment by maintaining a higher level of GSH [55]. In addition, a previous report suggested an intimate link between GSR and hepatocellular carcinoma malignancy [45]. However, the function of GSR in liver cancer and sorafenib resistance is poorly elucidated. Herein, our work was the first to clarify that GSR was important to maintain the redox state via GSH recycling in HCC and sorafenib resistance. GSR was highly expressed in liver cancer and closely associated with poor prognosis. Targeting GSR decreased GSH content and GSH/GSSG ratio, thereby sensitizing SLC27A5-KO cells to sorafenib-induced ferroptosis. BCNU, a selective inhibitor of GSR, markedly inhibited the enzyme activity of GSR and the effect was particularly remarkable in the liver tissues [56]. Importantly, the combination of sorafenib and BCNU has synergistic antitumor effects and thus enhanced the efficacy of ferroptotic cancer therapy both in vitro and in vivo. These results suggested that targeting GSR represents an effective strategy to perturb the redox balance, and GSR may be a potential therapeutic target for sorafenib-resistant HCC. However, this hypothesis should be evaluated in patients, which requires further study in the future.

In summary, this study uncovered a novel role of SLC27A5 in sorafenib resistance. SLC27A5 deficiency strengthens the endurance of sorafenib through activating the NRF2/GSR axis, which provides a potential breakthrough to reverse sorafenib resistance. GSR inhibitor BCNU amplifies sorafenib-induced ferroptosis. Altogether, our work suggests a promising strategy that induction of ferroptosis to sensitize HCC to sorafenib.

## Materials and methods

### Cell culture

Human HCC cell lines HepG2 and PLC/PRF/5 were acquired from the American Type Culture Collection (ATCC, VA, USA); SK-Hep1 cells were obtained from the Cell Bank of the Chinese Academy of Sciences (Shanghai, China). Cells were cultured with Dulbecco’s modified Eagle’s medium (DMEM; Hyclone, Logan, UT, USA) supplemented with 10% fetal bovine serum (FBS; Gibco, Rockville, MD, USA), 100 mg/ml of streptomycin, and 100 U/ml of penicillin in the incubator at 5% CO_2_, 37 °C, 95% humidity. All cell lines were tested for the absence of mycoplasma contamination.

### Adenovirus and Reporter plasmids

Adenoviruses overexpressing SLC27A5 (AdSLC27A5) and GFP control (AdGFP) were generated using the AdEasy system (Prof. T-C He, University of Chicago, USA) as previously described [27]. Human SLC27A5 expression plasmid was generated by subcloning PCR amplification and inserted into *Hind III* and *Sal I* sites of the pSEB-3Flag vector (Prof. T-C He, University of Chicago, USA).

### CRISPR/Cas9-mediated gene editing

Single guide RNA sequences targeting *SLC27A5* and *GSR* were designed (Table S1) using the E-CRISP (http://www.e-crisp.org/E-CRISP/designcrispr.html) and cloned into the Cas9-expressing lentiviral vector CRISPRv2 (Prof. Ding Xue, Tsinghua University, Beijing, China). Lentiviruses were packaged and stable KO cell lines were selected as previously described [27].

### Reagents and antibodies

Bafilomycin A1 (S1413) and ZVAD-FMK (S7023) were purchased from Selleckchem (Houston, TX, USA). N-acetyl-L-cysteine (S0077) was from the Beyotime Institute of Biotechnology (Shanghai, China). Sorafenib (HY-10201), Erastin (HY-15763), Ferrostatin-1 (HY-100579), Deferoxamine (HY-B0988), and Necrosulfonamide (HY100573) were obtained from MedChemExpress (MCE; Shanghai, China). Tertiary butylhydroquinone (112941) was purchased from Sigma–Aldrich (Shanghai, China). Brusatol (MB7292) was obtained from Meilunbio (Dalian, China). Carmustine (BCNU) was from Macklin (Shanghai, China). Antibodies against SLC27A5 (NBP1-89267) were brought from Novusbio (Centennial, CO, USA). Anti-NRF2 (ab62352), anti-β-actin (ab6276), and anti-4-HNE (ab46545) were obtained from Abcam (Cambridge, MA, USA). Anti-GSR (sc-133245) was purchased from Santa Cruz Biotechnology (Santa Cruz; CA, USA).

### Clone formation assay

Cells were planted in the six-well plate (2 × 10^3^ cells per well) and cultivated at 37 °C for 2 weeks with DMEM mixed with 10% serum. The culture medium containing either DMSO or sorafenib (10 μM) was replaced every 72 h. Colonies were washed using PBS, followed by fixation with 4% paraformaldehyde for 30 min, and stained with 0.004% crystal violet. After being photographed, colonies were counted using ImageJ.

### Cell growth curve

Cell proliferation was determined by the IncuCyte ZOOM Live-Cell Imaging system (Essen BioScience, MI, USA). Cells were seeded in 96-well format (1 × 10^3^ cells per well) and cultured with drugs for 4 days. The cells images were scanned every 24 h and the growth curve was plotted based on the area of the cells which was generated by IncuCyte ZOOM software.

### Cell viability assay

For cell viability assay, the Cell Counting Kit-8 (CCK-8) (Topscience, Shanghai, China) was prepared to assess cell viability following the manufacturer’s instructions. One thousand cells/well were plated in 96-well plates and incubated at 37 °C. Cells were exposed to the drugs for 24 h continuously, after which cell viability was assayed by microplate reader (Thermo Fisher Scientific, MA, USA) at 450 nm absorbance. Cell viability was calculated as:

$${\mathrm{Cell}}\;{\mathrm{viability}}\left( \% \right) = \left( {{\mathrm{OD}}_{450({\mathrm{experimental}}\;{\mathrm{group}})} - {\mathrm{OD}}_{450({\mathrm{blank}}\;{\mathrm{group}})}/{\mathrm{OD}}_{450({\mathrm{control}}\;{\mathrm{group}})} - {\mathrm{OD}}_{450({\mathrm{blank}}\;{\mathrm{group}})}} \right) \times 100\%$$

The black group contained only the medium and cells in the control group were cultured with DMSO. All cell-based assays were completed at least in triplicate.

### Half maximal inhibitory concentration assay

Indicated cells were planted on 96-well plate (1 × 10^4^ cells per well). The various concentrations of sorafenib were cultured with cells for 24 h. After 24 h, sorafenib sensitivity was monitored at 450 nm by a microplate reader (Thermo Fisher Scientific). The half-maximal inhibitory concentration (IC_50_) was acquired by nonlinear regression in GraphPad Prism (version 8.0).

### ROS measurement

Cells planted on coverslips or fresh frozen tissue sections were incubated with the CellROX Deep Red Reagent (C10422, Life Technologies, CA, USA) at a final concentration of 5 μM for 30 min at 37 °C. Washed coverslips were fixed with 4% paraformaldehyde for 15 min at room temperature. DAPI staining for nuclear labeling was performed and then Analyzing the signal within 2 h using confocal microscope (Leica TCS SP8, Solms, Germany). Mean fluorescence intensity was qualified by ImageJ. Alternatively, the indicated cells or fresh frozen tissue sections were incubated with DCFH-DA probe (S0033, Beyotime) for 30 min at 37 °C. The excess unbound probe was eliminated with serum-free medium followed by flow cytometric analysis or fluorescence observation.

### Lipid peroxidation analysis

For analysis of lipid peroxidation, cells were plated at an appropriate density, treated with the indicated compound, and then stained with BODIPY-C11 (D3861, Invitrogen, USA) for 30 min at 37 °C. The collected cells were resuspended in PBC containing 5% FBS. The fluorescence signal of FITC and phycoerythrin channel were read using flow cytometry or confocal microscope.

### Detection of malondialdehyde (MDA)

The level of malondialdehyde, an indicator of lipid peroxidation, was quantified using Lipid Peroxidation MDA Assay Kit (S0131, Beyotime) following the manufacturer’s instructions. In Brief, malondialdehyde reacted with thiobarbituric acid (TBA) to form red products, which were detected by colorimetric assay.

### Measurement of glutathione

The intracellular and tissue reductive GSH and oxidative GSH (GSSG) levels were detected by GSH and GSSG Assay Kit (S0053, Beyotime). Cells were lysed by repeated freezing and thawing. Then collected the supernatant after centrifugation to measure GSH and GSSG based on the manufacturer’s protocol.

### Determination of GSR activity

The enzyme activity of GSR was analyzed using a GSR assay kit (S0055, Beyotime). Prepared cells were washed twice and lysed with a cell lysate. Extracted GSR catalyzes the reduction of GSSG to GSH upon the consumption of NADPH in the reaction system. GSH reacts with DTNB and the absorbance of the yellow product was determined at 412 nm.

### Iron assay

The relative labile iron was determined using Iron Assay Kit (Sigma, MAK025). Samples were lysed in 4 times volumes of lysis buffer. The prepared supernatants reacted with Assay Buffer and Iron Reducer for 30 min. And then, Iron Probe was added to the reaction system. After incubation for 60 min, the absorbances were detected.

### RNA isolation and quantitative reverse-transcription PCR

Total RNA was extracted from mice liver tissues and HCC cell lines using RNAiso plus reagent (9108, Takara, Japan). Purified RNA was reverse-transcribed into cDNA using the PrimeScript™ RT reagent kit (RR047A, Takara). Quantitative real-time PCR was conducted with the SYBR Green qPCR Master Mix (S2014, US EVERBRIGHT, Suzhou, China) with specific primers (Table S1). The target transcript level was quantified based on the 2^−ΔΔCT^ value normalized the β-actin.

### Western blotting analysis

Protein samples of HCC cells or liver tissues were extracted and quantified as previously described [57]. Equal volumes of samples were fractionated by SDS-PAGE and transferred onto PVDF membranes (Millipore, Billerica, MA, USA). Then membranes were blocked with 5% skimmed milk and incubated with primary antibodies overnight at 4 °C. After incubation with peroxidase-conjugated secondary antibodies, signals were visualized by enhanced Chemiluminescence substrate Kits (ECL, New Cell & Molecular Biotech Co, Ltd, China).

### Transmission electron microscope (TEM)

Cell clumps obtained from centrifuged samples were fixed with 2.5% glutaraldehyde overnight at 4 °C. After being fixed with 2% tetroxide, samples were dehydrated in a graded ethanol series and embedded in epoxy resin. Thin sections were imaged by Hitachi-7500 transmission electron microscope (Hitachi, Tokyo, Japan).

### Immunohistochemistry (IHC)

Paraffin-embedded liver specimens were deparaffinized at 55 °C for 2 h and rehydrated through gradient alcohol followed by antigen retrieval performed in a pressure cooker with citrate buffer (pH 6.0). Subsequently, endogenous peroxidase was quenched by pretreatment with 3% H_2_O_2_ for 30 min at room temperature. Sections were incubated with corresponding primary antibodies overnight at 4 °C and secondary antibodies for 1 h at room temperature. Sections were stained with DAB and counterstained with hematoxylin. Images of stained slides were obtained using Pannoramic Viewer 1.15.2 (3DHistech, Budapest, Hungary).

### Animal model and treatment

Age-matched male BALB/c nude mice (4–6 weeks old) were used for the orthotopic mouse model. Cohorts of mice were randomized into different treatment groups. 4 × 10^6^ tumor cells were suspended in a 50 µl PBS/Matrigel (356234, BD Biosciences) mixture (1:1 (v/v) ratio) for each group of mice and injected into the left liver lobes by surgical implantation. An overview of the animal model is presented (Fig. 7A).

### Statistical analysis

All quantitative data were presented as means ± standard deviation (SD). Appropriate statistical analyses were performed using GraphPad Prism 8.0 software (GraphPad Software Inc, La Jolla, CA, USA).

## Supplementary information

supplementary material

original western blot

author contribution

checklist

## Data Availability

The data that support the findings of this study are available from the corresponding author upon reasonable request.
