# Supplementary material for: SLC27A5 promotes sorafenib-induced ferroptosis in hepatocellular carcinoma by downregulating glutathione reductase
Source: Cell Death Dis. 2023 Jan 12;14(1):22. doi: 10.1038/s41419-023-05558-w (PMC9837139; doi:10.1038/s41419-023-05558-w)
Supplement: Supplementary file 1 — supplementary material [file 41419_2023_5558_MOESM1_ESM.docx]

**Supplementary Materials for**

SLC27A5 promotes sorafenib-induced ferroptosis in hepatocellular carcinoma by downregulating glutathione reductase

Feng-li Xu^#^, Xiao-hong Wu^#^, Chang Chen, Kai Wang, Lu-yi Huang, Jie Xia, Yi Liu, Xue-feng Shan*, Ni Tang*

*Corresponding author. Email: shanxuefeng@hospital.cqmu.edu.cn (X.S.), nitang@cqmu.edu.cn (N.T.)

Contents

Supplementary table, figures and legends

Table S1. Primer sequences used in this study

Table S2. Patient information of 10 HCC samples with IHC staining.

Figure S1. SLC27A5 expression is downregulated in public dataset from sorafenib-resistant HCC cells.

Figure S2. Loss of SLC27A5 enhances the resistance of HCC cells to sorafenib.

Figure S3. SLC27A5-dependent sorafenib resistance was mediated by ferroptosis in HCC.

Figure S4. SLC27A5 overexpression overcomes the resistance of HCC-SR cells to sorafenib by inducing ferroptosis.

Figure S5. SLC27A5 negatively regulates NRF2/GSR signaling pathway in HCC.

Figure S6. Silencing GSR sensitizes HCC cells to sorafenib-induced ferroptosis.

Figure S7. BCNU combination enhances the curative effect of sorafenib in vivo.

| Name | Sense Primer (5’-3’) | | Antisense Primer (5’-3’) | |  |
| --- | --- | --- | --- | --- | --- |
| Primers for sub-clone | | | | |  |
| *SLC27A5*  (Human) | | CGGAAGCTT ATGGGTGTCAGGCAACAGTTGGCCTTG | | CATGTCGAC GAGCCTCCAGGTTCCCTCACA | |
| Primers for real-time PCR | | | | |  |
| *SLC27A5*  (Human) | | GAGGGCAGAATCATCACGAAG | TGTGCTGTAGGAAGCTCATCTCTC | |  |
| *ACSL4*  (Human) | AAACCAAAGAACACCATTGCC | | GCCTCAGATTCATTTAGCCCAT | |  |
| *POR*  (Human) | AAACGGGGAGGAACATCATC | | GCATTGTCGGTGGGGTCT | |  |
| *TFR1*  (Human) | GCTTTCCCTTTCCTTGCATAT | | CACGAACTGACCAGCGACCT | |  |
| *NCOA4*  (Human) | CCCTTTTGAGGTGTAGTGATGC | | TGAGCCTGCTGTTGAAGTGTC | |  |
| *SLC7A11*  (Human) | TTTCTGAGCGGCTACTGGG | | CAAAGGGTGCAAAACAATAACA | |  |
| *GPX4*  (Human) | CCGCCTTTGCCGCCTAC | | TTTACTTCGGTCTTGCCTCACT | |  |
| *GCLC*  *(Human)* | GGCGATGAGGTGGAATACAT | | GTCCTTTCCCCCTTCTCTTG | |  |
| *GCLM*  *(Human)* | GACAAAACACAGTTGGAACAGC | | CAGTCAAATCTGGTGGCATC | |  |
| *GSS*  *(Human)* | GGCTGAGGGAGTATTGCTGA | | TTGATGGTGCTGGAAAGAGTT | |  |
| *GSR*  *(Human)* | AAAAGCGGGATGCCTATGT | | GATGTGTGGGGCGGTGTA | |  |
| *TXNRD1*  *(Human)* | ACACAAAGCTTCAGCATGTCA | | CAATTCCGAGAGCGTTCC | |  |
| *HO-1*  *(Human)* | AACTTTCAGAAGGGCCAGGT | | CTGGGCTCTCCTTGTTGC | |  |
| *β-actin*  (Human) | AGGCCAACCGCGAGAAGATGACC | | GAAGTCCAGGGCGACGTAGCAC | |  |
| Primers for sgRNA | | | | |  |
| *GSR sgRNA1*  (Human) | CACCGTACACATCCAACATTCACC | | AAACGGTGAATGTTGGATGTGTAC | |  |
| *GSR sgRNA2*  (Human) | CACCGATCATGCATGAATTCAGAG | | AAACCTCTGAATTCATGCATGATC | |  |

**Table S1.** Primer sequences used in this study.

| order | sex | age | Alpha  fetoprotein (ng/mL) | Hepatitis B  surface antigen | Histological  differentiation | Recurrence |
| --- | --- | --- | --- | --- | --- | --- |
| 1 | Female | 50 | 5.43 | (+) | poor | present |
| 2 | Male | 36 | 22.8 | (+) | poor | present |
| 3 | Male | 44 | 246.6 | (+) | middle | present |
| 4 | Male | 63 | 1111.5 | (+) | well | present |
| 5 | Female | 41 | 2.35 | (-) | poor | absent |
| 6 | Male | 68 | 2564 | (-) | poor | absent |
| 7 | Male | 43 | 1642 | (+) | middle | absent |
| 8 | Female | 35 | 1362.9 | (+) | poor | present |
| 9 | Male | 59 | 668.2 | (+) | middle | present |
| 10 | Male | 66 | 7285.1 | (+) | well | present |

**Table S2.** Patient information of 10 HCC samples with IHC staining.


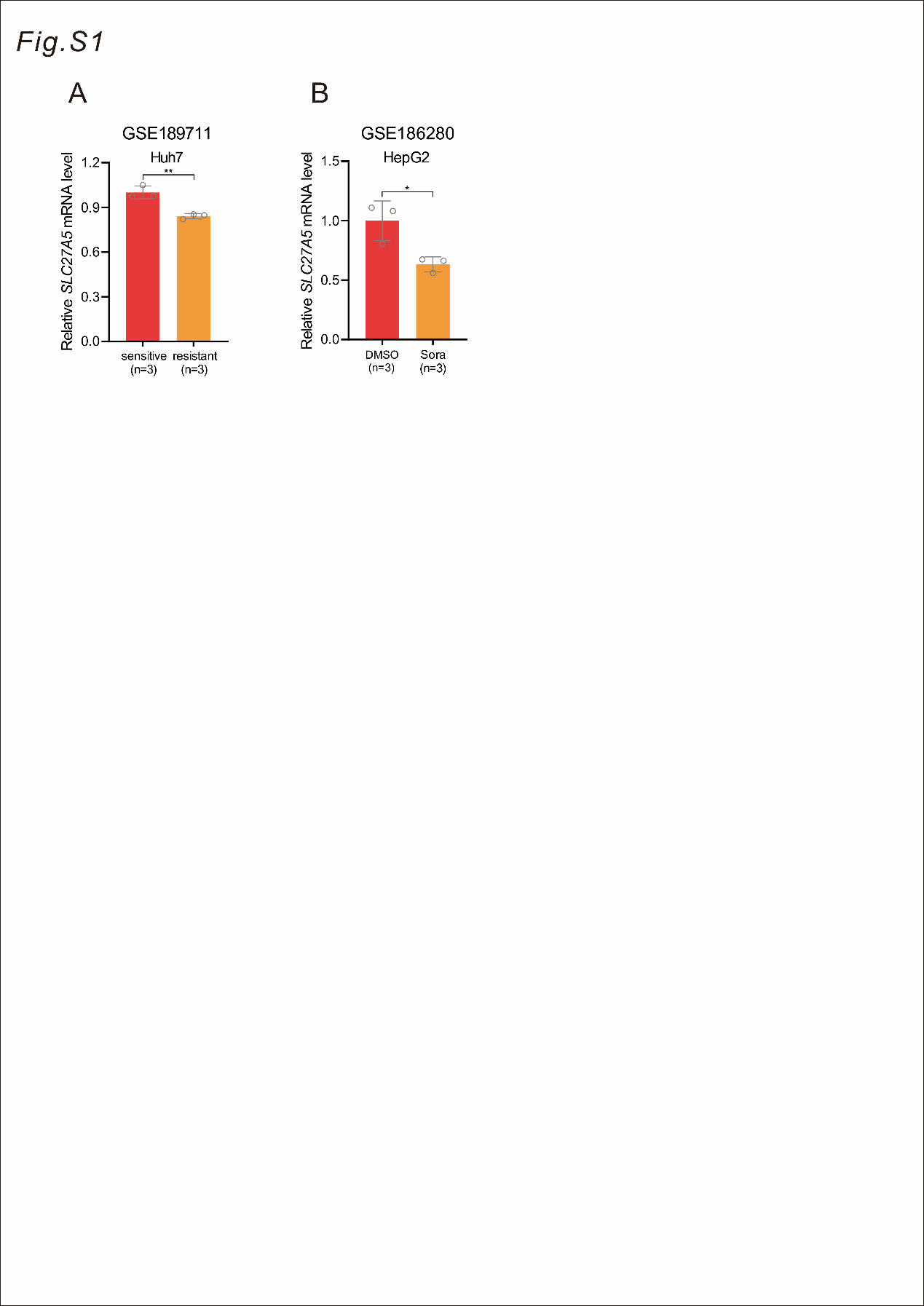


**Figure S1. SLC27A5 expression is downregulated in public dataset from sorafenib-resistant HCC cells.** **A** *SLC27A5* mRNA levels between sorafenib-resistant Huh7 cells (n=3) and sorafenib-resistant HepG2 cells (n=3). **B** The changed mRNA level of *SLC27A5* in HepG2 with sorafenib treatment (n=3) compared with DMSO treatment (n=3).


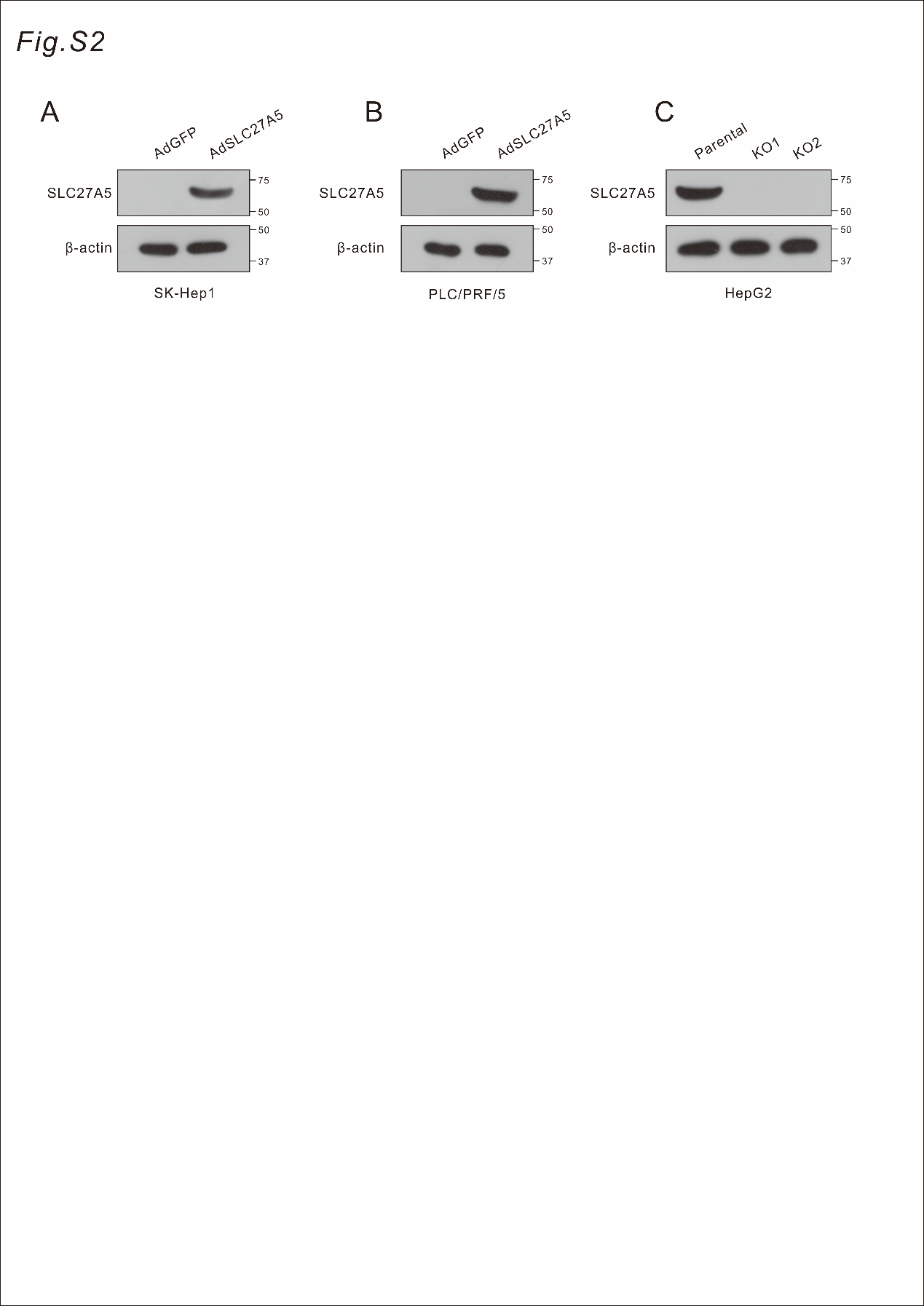


**Figure S2. Loss of SLC27A5 enhances the resistance of HCC cells to sorafenib.** **A**, **B** ectopic expression of SLC27A5 using the AdEasy system was confirmed in SK-Hep1 and PLC/PRF/5 cells. **C** Knockout of SLC27A5 in HepG2 cells was confirmed by Western blot.


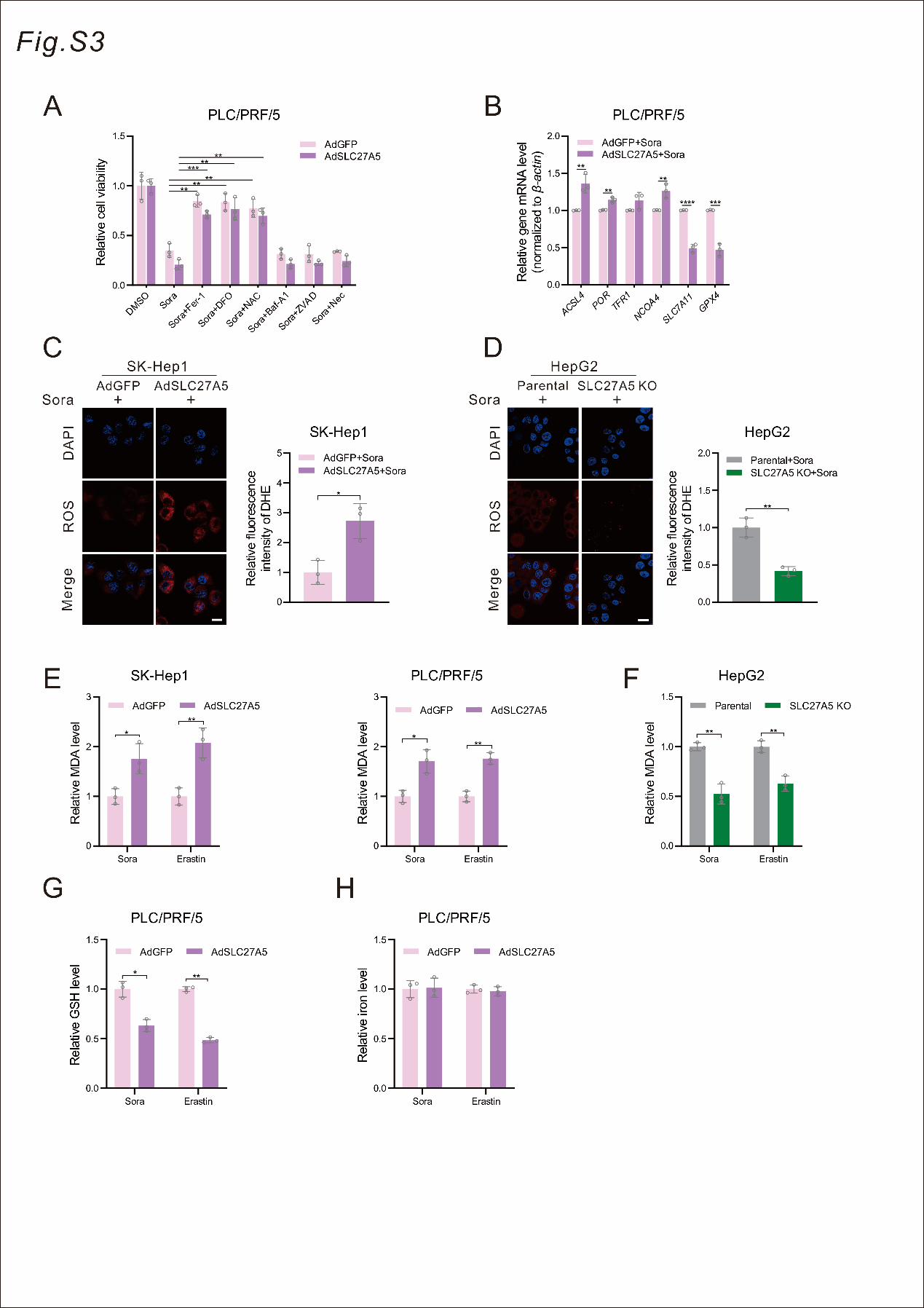


**Figure S3. SLC27A5-dependent sorafenib resistance is mediated by ferroptosis in HCC. A** SLC27A5-overexpression PLC/PRF/5 cells were incubated with sorafenib (10 μM) alone or co-treatment with sorafenib and anyone cell death inhibitor for 24h and cell viability was determined. **B** The relative mRNA level of biomarker genes related to ferroptosis by RT-qPCR in PLC/PRF/5. **C**, **D** Typical immunofluorescence images (left) and quantification (right) of reactive oxygen species (ROS) level in SLC27A5-overexpression (**C**) and SLC27A5-KO (**D**) HCC cells. Scale bar: 20 μm. **E**, **F** The intracellular MDA levels in SLC27A5-overexpression SK-Hep1 PLC/PRF/5 (**E**) and SLC27A5-KO HepG2 (**F**). **G**, **H** Levels of GSH (**G**) and Fe^2+^ (**H**) were determined in SLC27A5-overexpressing cells. Values represent the mean ± SD (n = 3). Statistical significance was calculated using two-tailed unpaired Student’s t-test and one-way ANOVA test. *p < 0.05, **p< 0.01 ***p< 0.001.


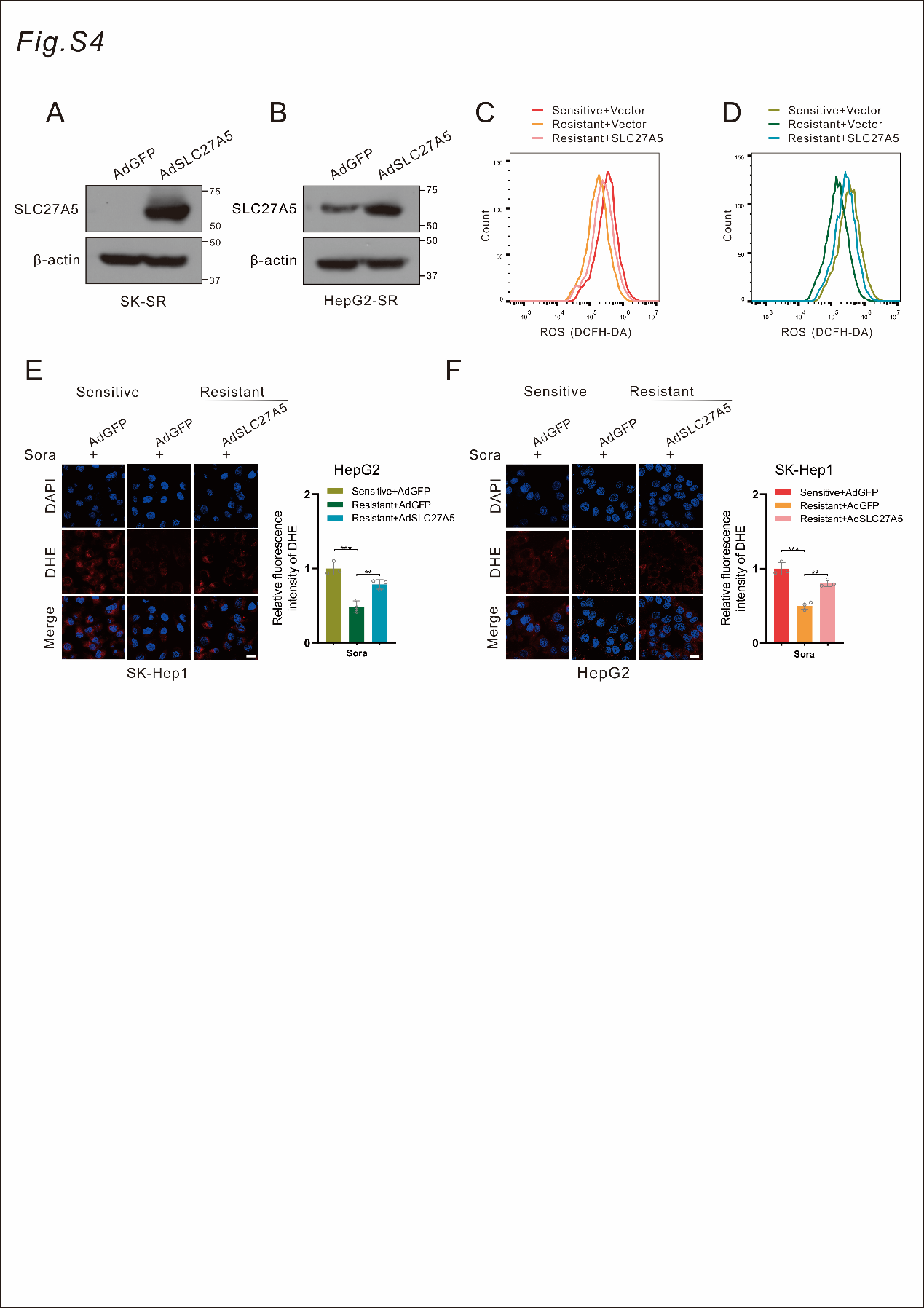


**Figure S4. SLC27A5 overexpression overcomes the resistance of HCC-SR cells towards sorafenib by inducing ferroptosis. A**, **B** The overexpression efficacy of SLC27A5 was identified by western blot analysis in sorafenib-resistant SK-Hep1 (**A**) and HepG2 (**B**). **C**, **D** Flow cytometry was performed to analyze the relative ROS level in SK-Hep1-SR and HepG2-SR cells transfected with pSEB-3Flag-SLC27A5 plasmid. **E**, **F** Representative fluorescence microscopic images (left) and quantification (right) of sorafenib-resistant SK-Hep1 (**E**) and HepG2 (**F**). scale bar: 20 μm.


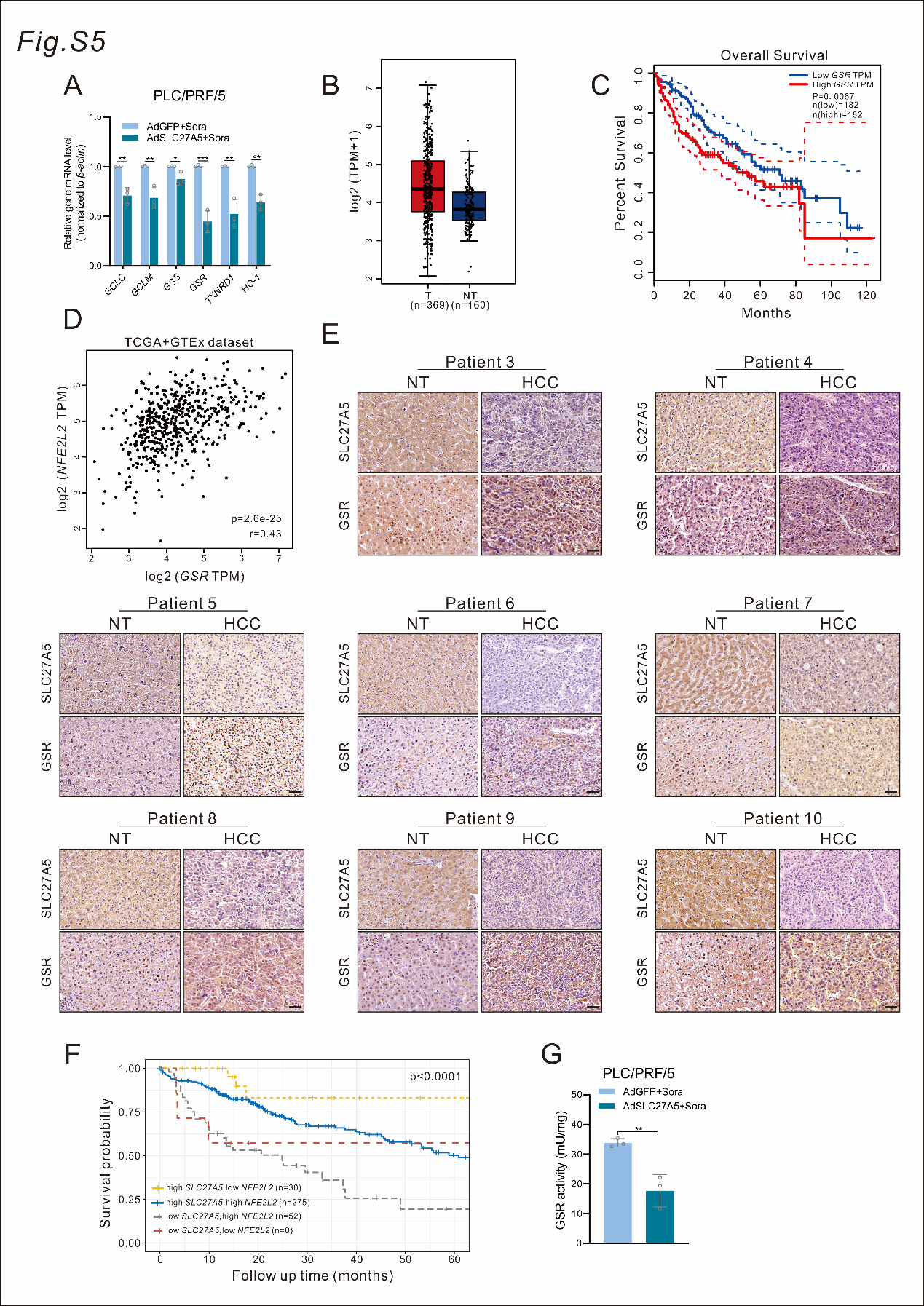


**Figure S5. SLC27A5 negatively regulates NRF2/GSR signaling pathway in HCC. A** Representative mRNA level of NRF2 downstream gene expression in SLC27A5-overexpression PLC/PRF/5 cultured with sorafenib for 24 h. **B**, **C** Differential expression (**B**) and prognostic analysis (**C**) of *GSR* in liver cancer using the GEPIA analysis tool (http://gepia.cancer-pku.cn). **D** Correlation analysis of the mRNA levels of *NFE2L2* and *GSR* in the liver by spearman. **E** Histochemical staining for SLC27A5 and GSR in HCC tissues and adjacent non-cancerous tissues. Scale bar: 50 μm. **F** Kaplan-Meier survival curve analysis based on the expression of *SLC27A5* and *NFE2L2* in the liver tumor. **G** The enzyme activity of GSR in SL27A5-overexpression PLC/PRF/5. Values represent the mean ± SD (n = 3). Statistical significance was calculated using two-tailed unpaired Student’s t-test. *p < 0.05, **p< 0.01, ***p< 0.001.


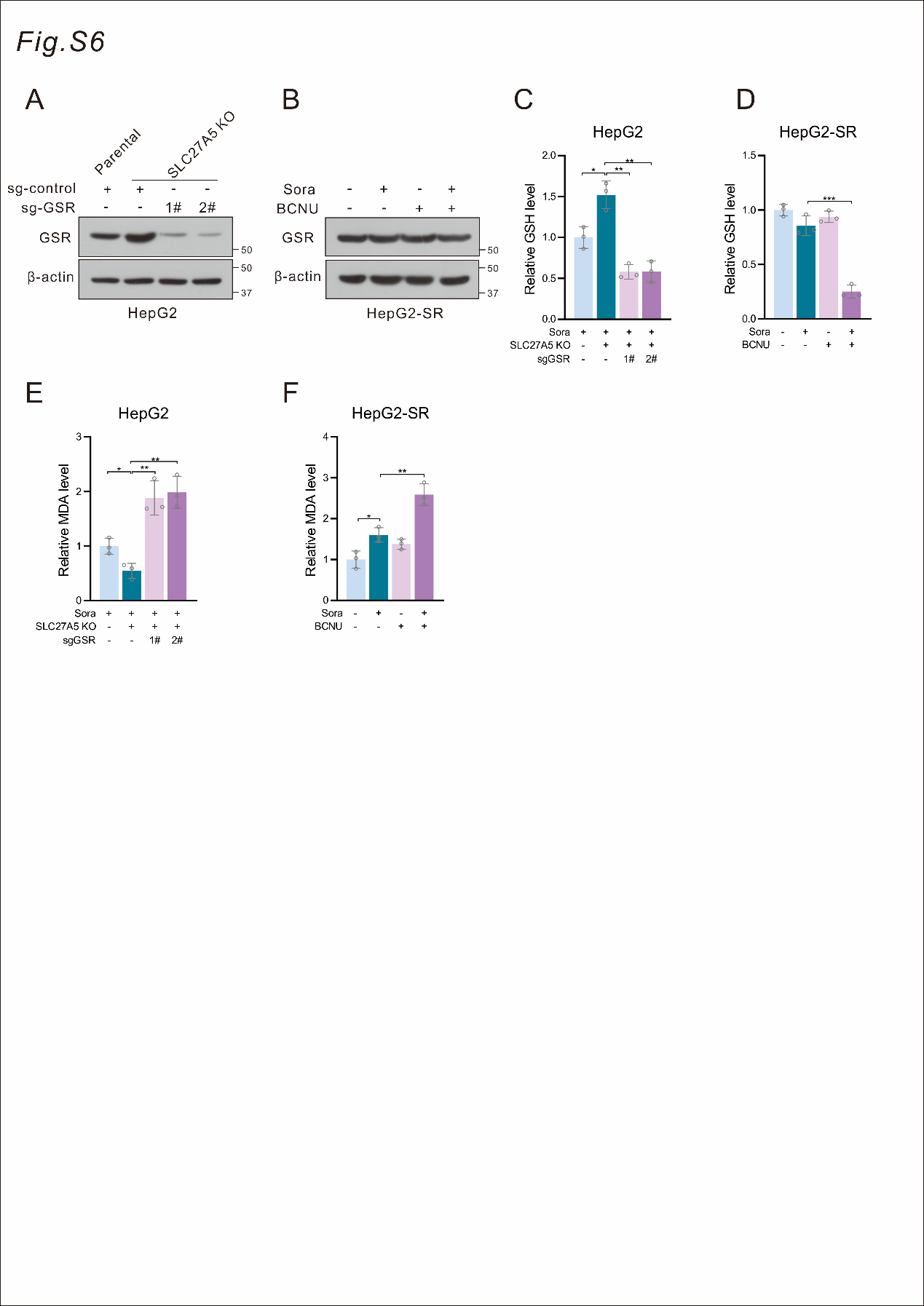


**Figure S6. Silencing GSR sensitizes HCC cells to sorafenib-induced ferroptosis. A**, **B** The protein expression of GSR in HepG2 transfected with sg*GSR* (**A**) and HepG2-SR treated with sorafenib or/and BCNU (**B**). **C**-**F** The relative GSH (**C**, **D**) and MDA level (**E**, **F**) in HepG2 transfected with sgGSR and HepG2-SR treated with sorafenib or/and BCNU. Quantitative data are represented as the mean ± SD (n=3). Statistical significance was calculated using one-way ANOVA test. **p< 0.01 ***p< 0.001.


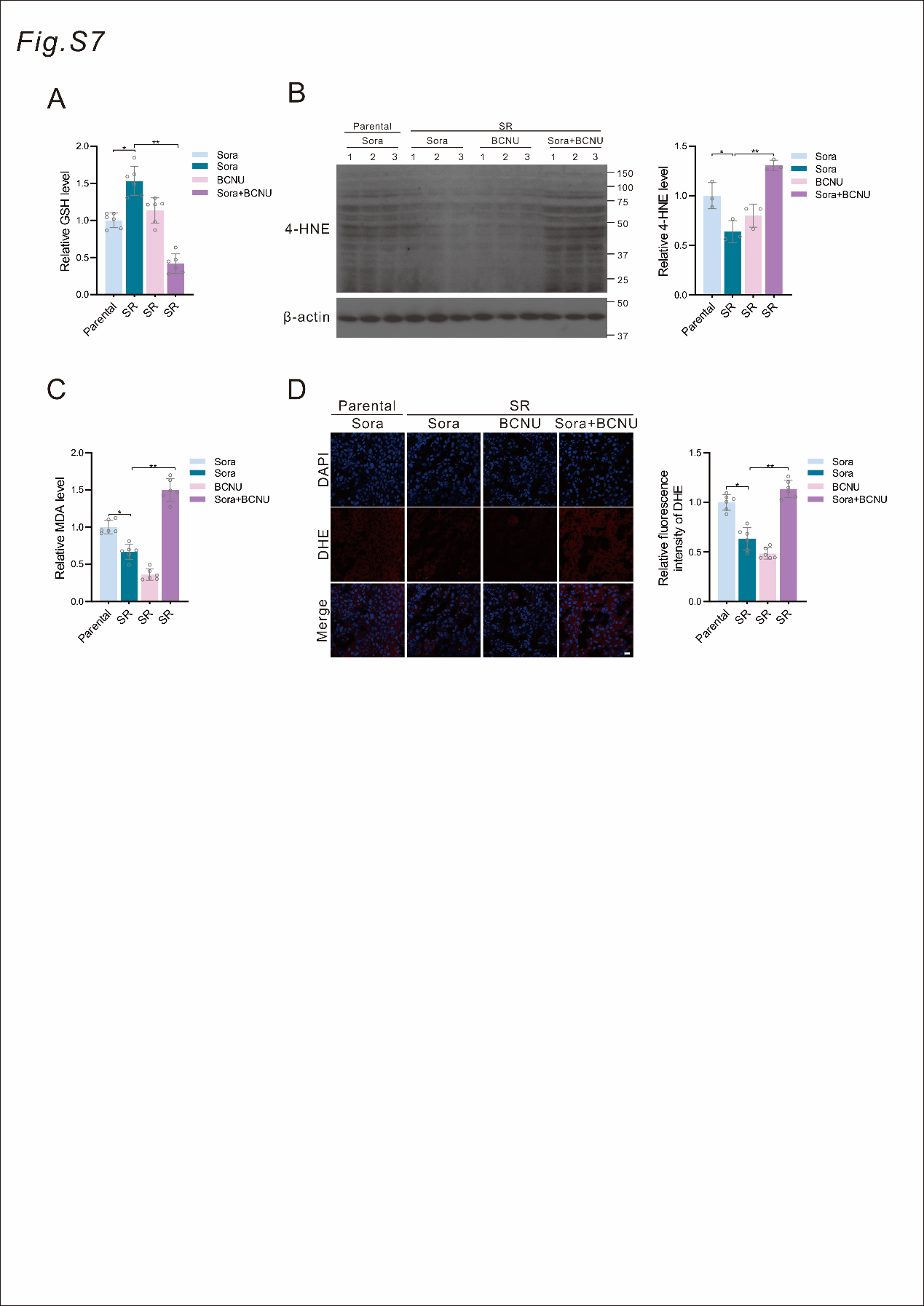


**Figure S7. BCNU combination enhances the curative effect of sorafenib in vivo. A**, **B** The levels of GSH (**A**) (n=6), 4-HNE-induced protein modification (**B**) (n=3) and MDA (**C**) (n=6) in tumor tissues were assayed. **D** Typical fluorescence microscopic images of tumor tissues stained with DHE probe. Scale bar: 10 μM. Values represent the mean ± SD. Statistical significance was calculated using one-way ANOVA test. *p < 0.05·, **p< 0.01 ***p< 0.001.
