# Supplementary figures and images for: SLC27A5 promotes sorafenib-induced ferroptosis in hepatocellular carcinoma by downregulating glutathione reductase
Source: Cell Death Dis. 2023 Jan 12;14(1):22. doi: 10.1038/s41419-023-05558-w (PMC9837139; doi:10.1038/s41419-023-05558-w)

Fig1D

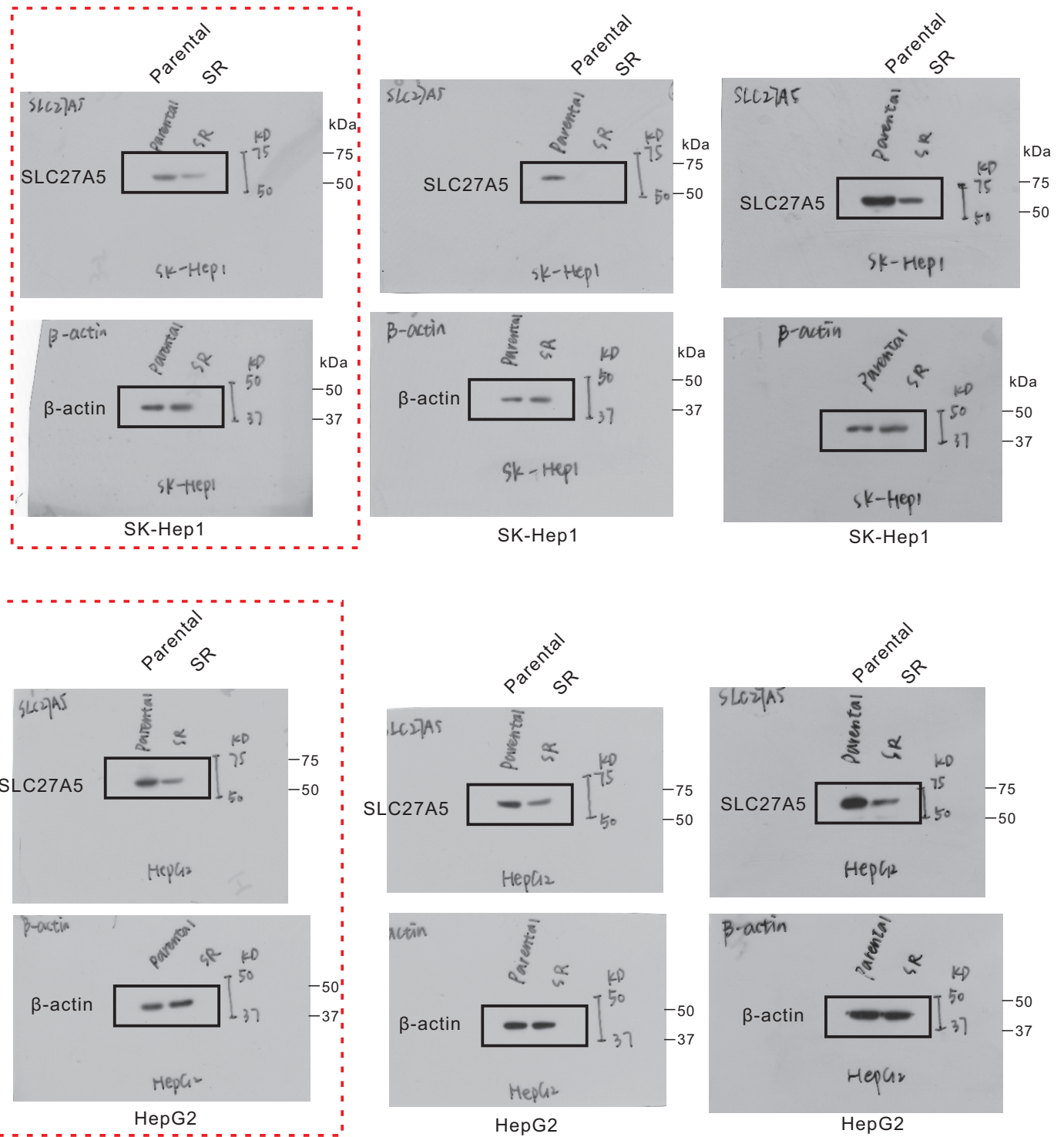

Fig4D

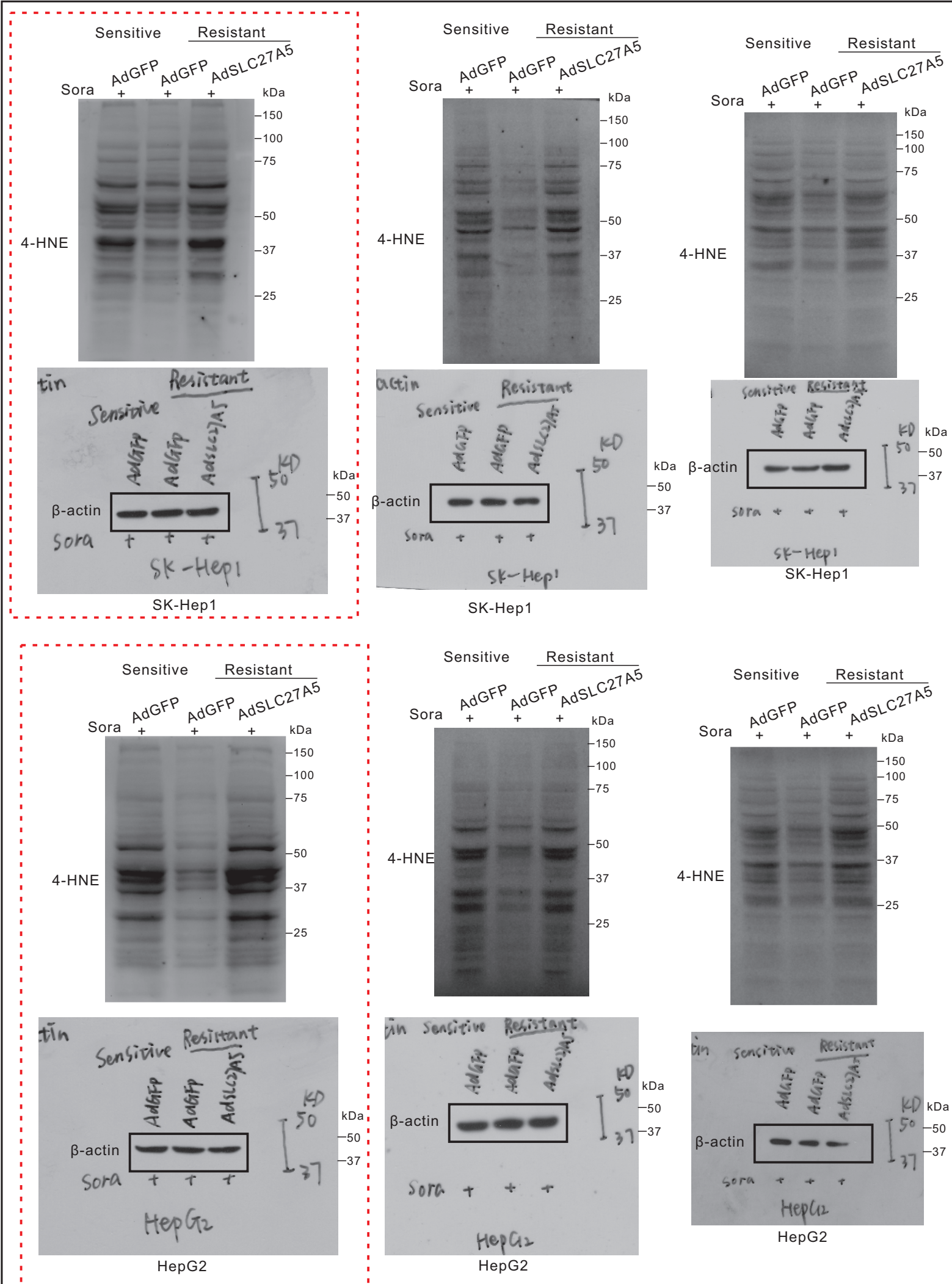

Fig5F

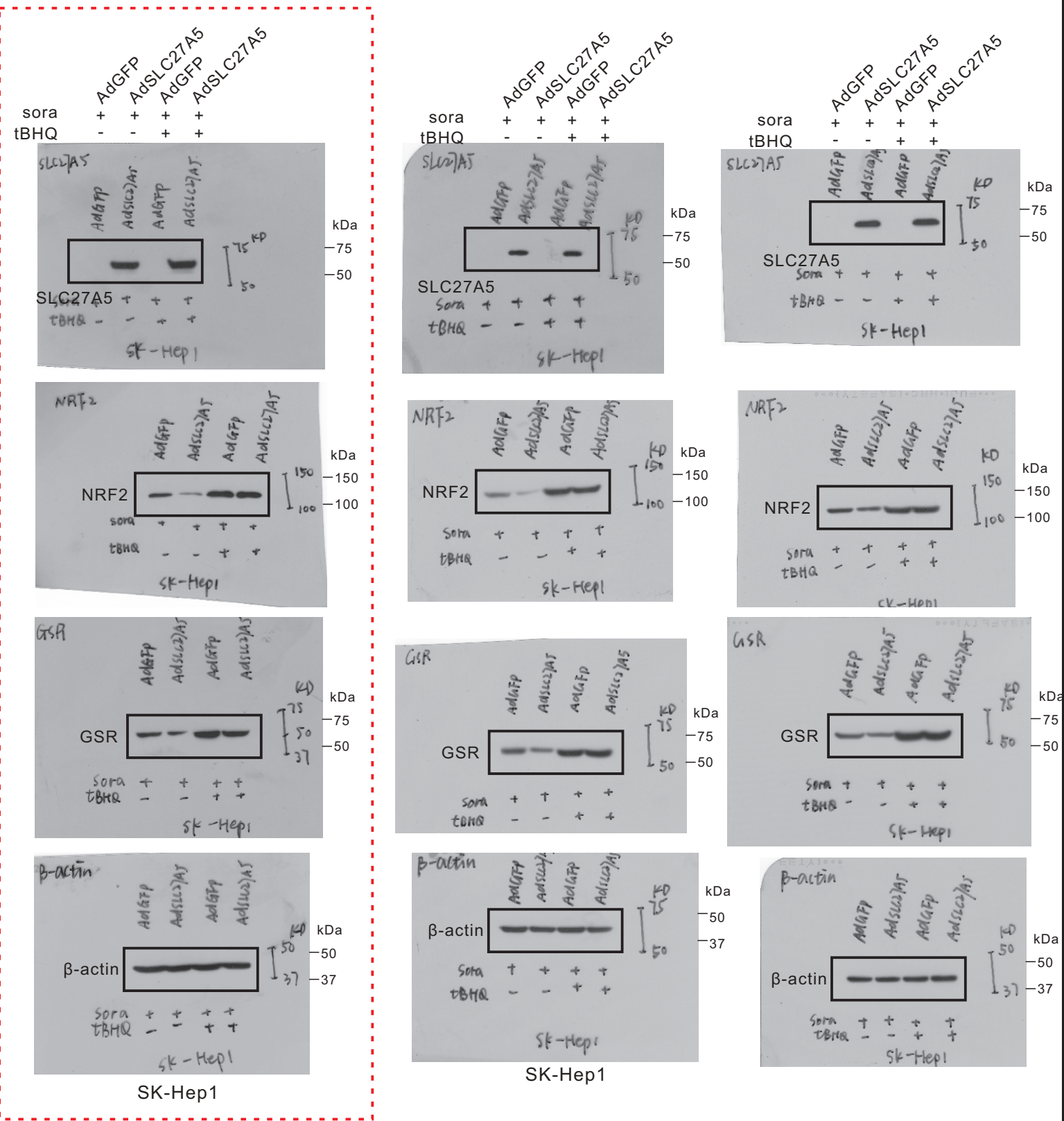

Fig5G

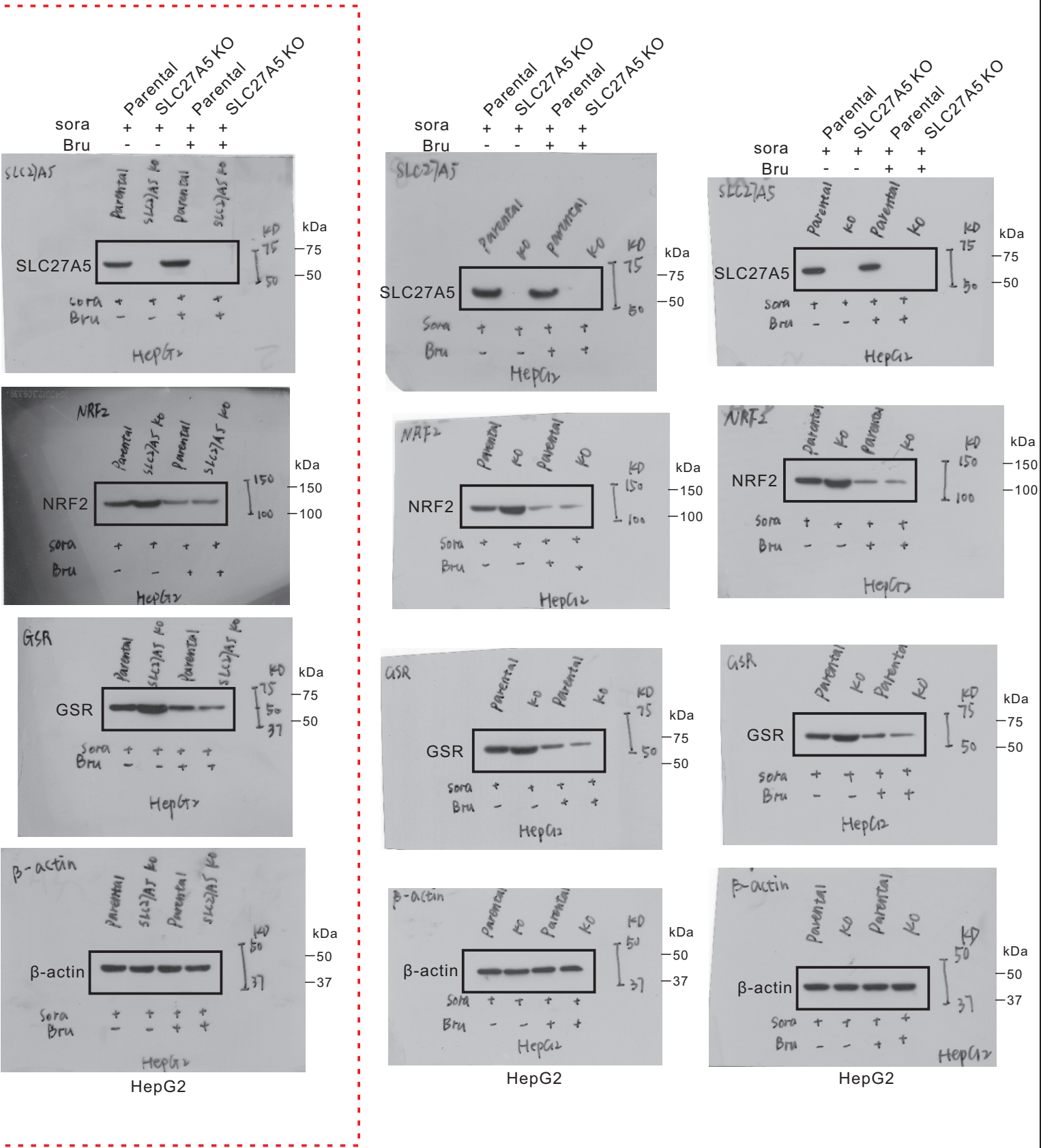

Fig6E

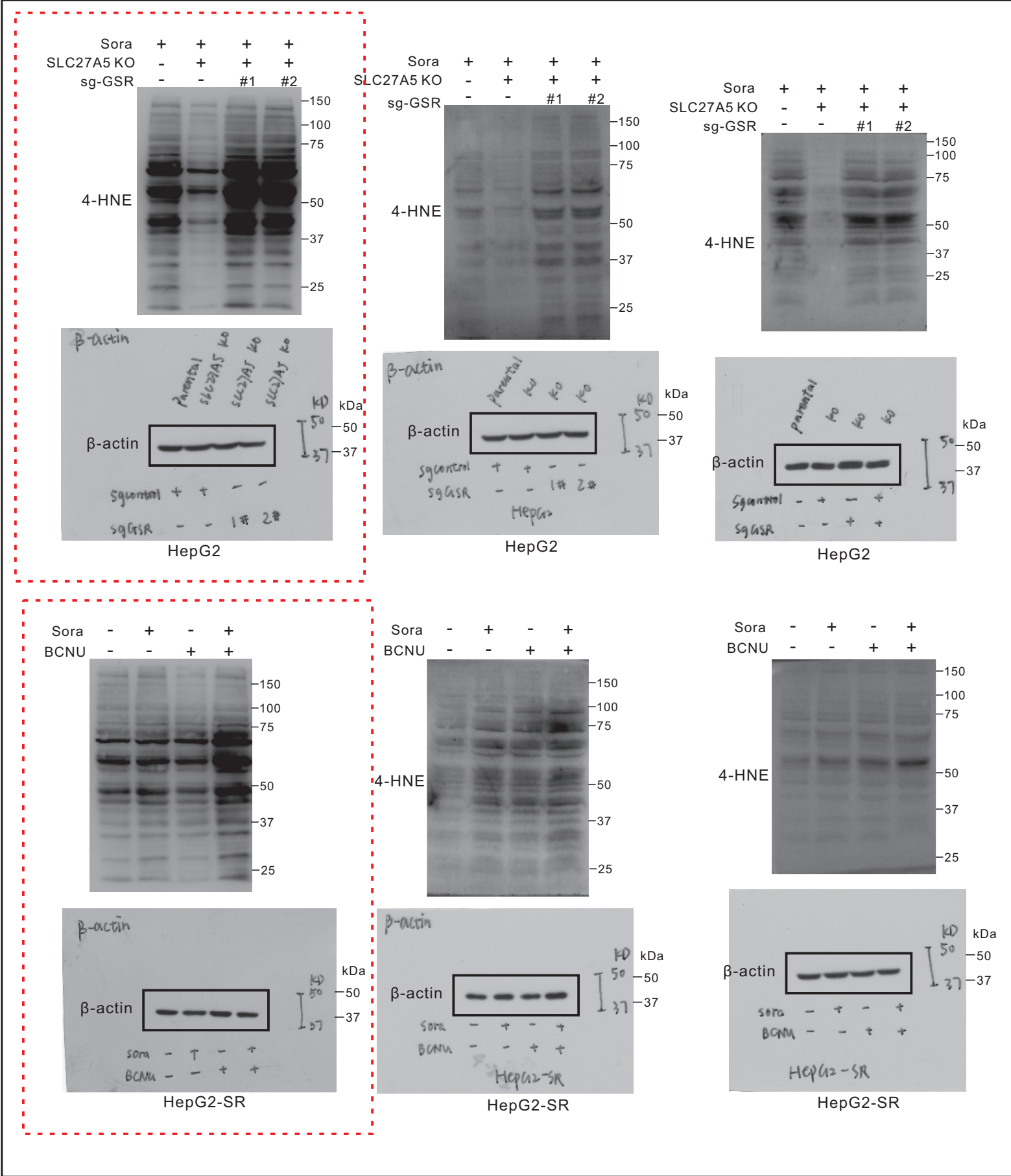

Fig7F

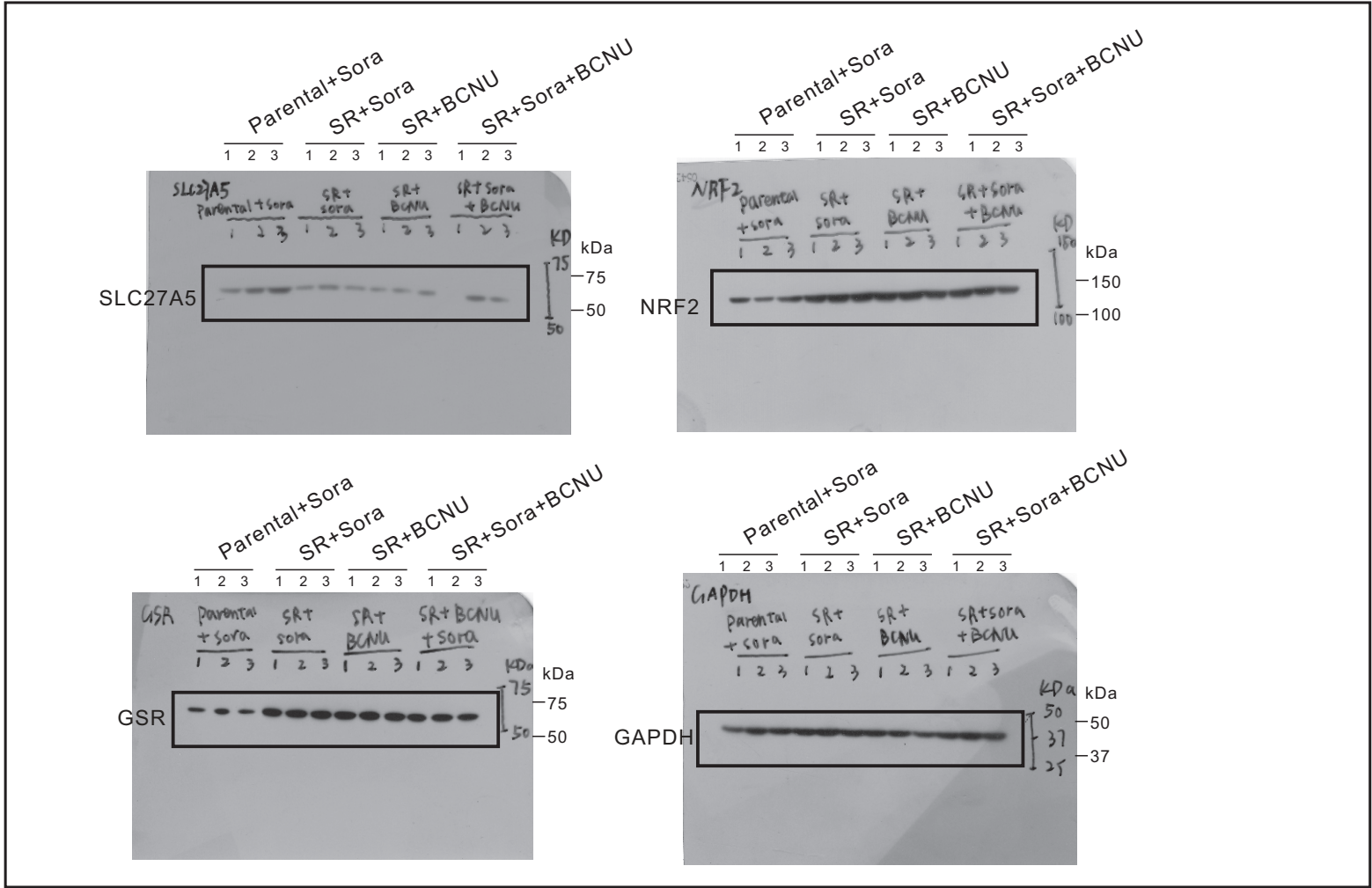

FigS2A

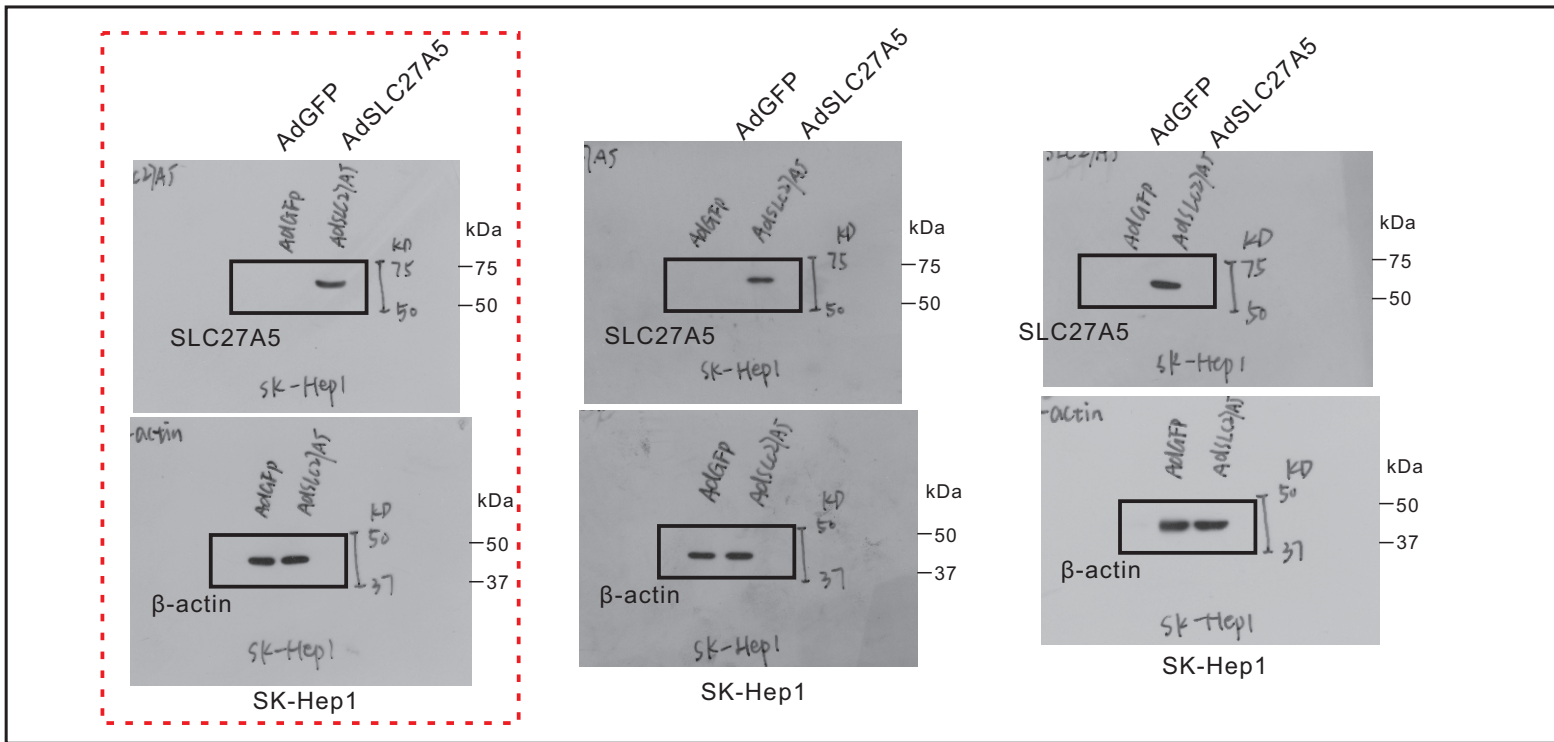

FigS2B

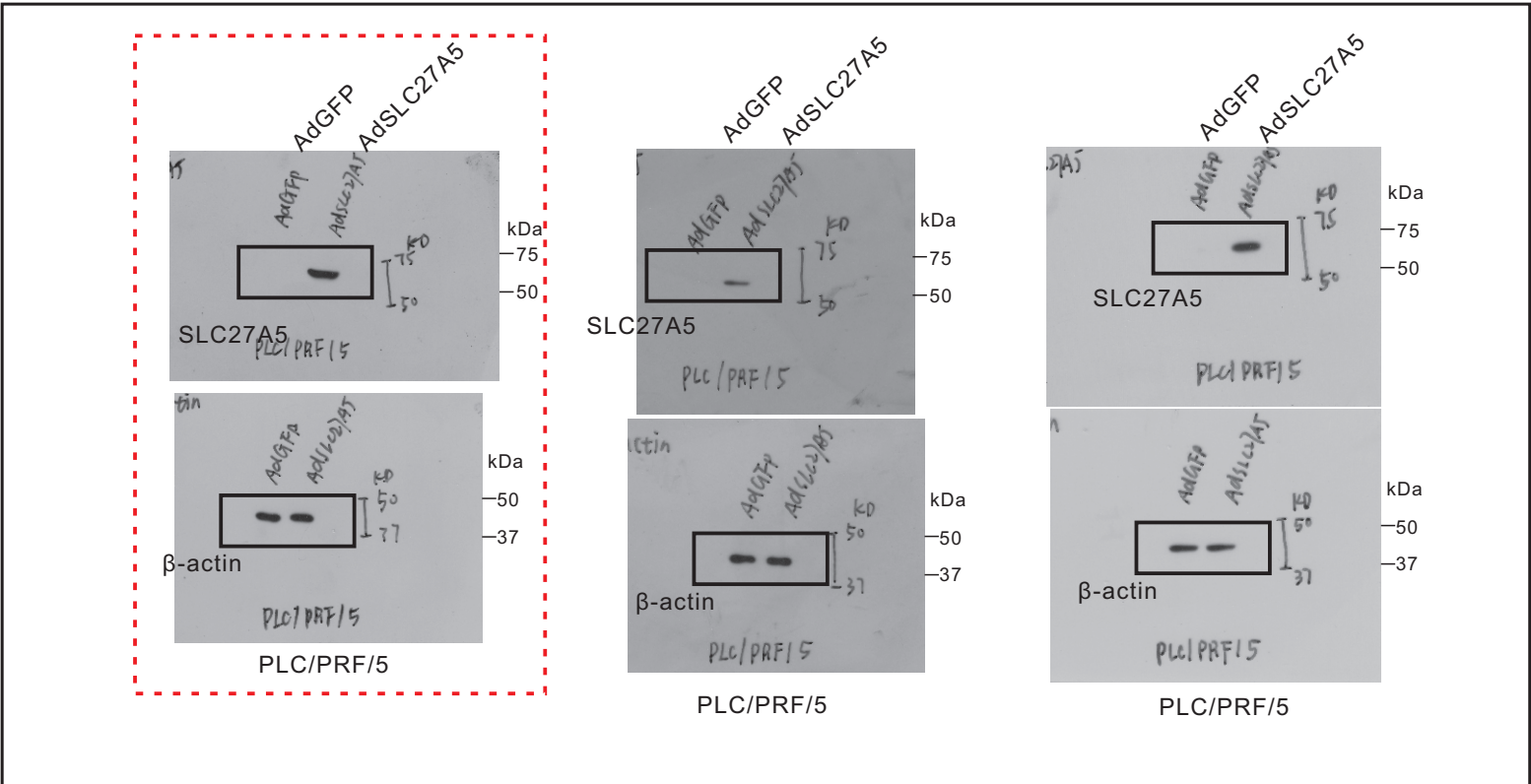

FigS2C

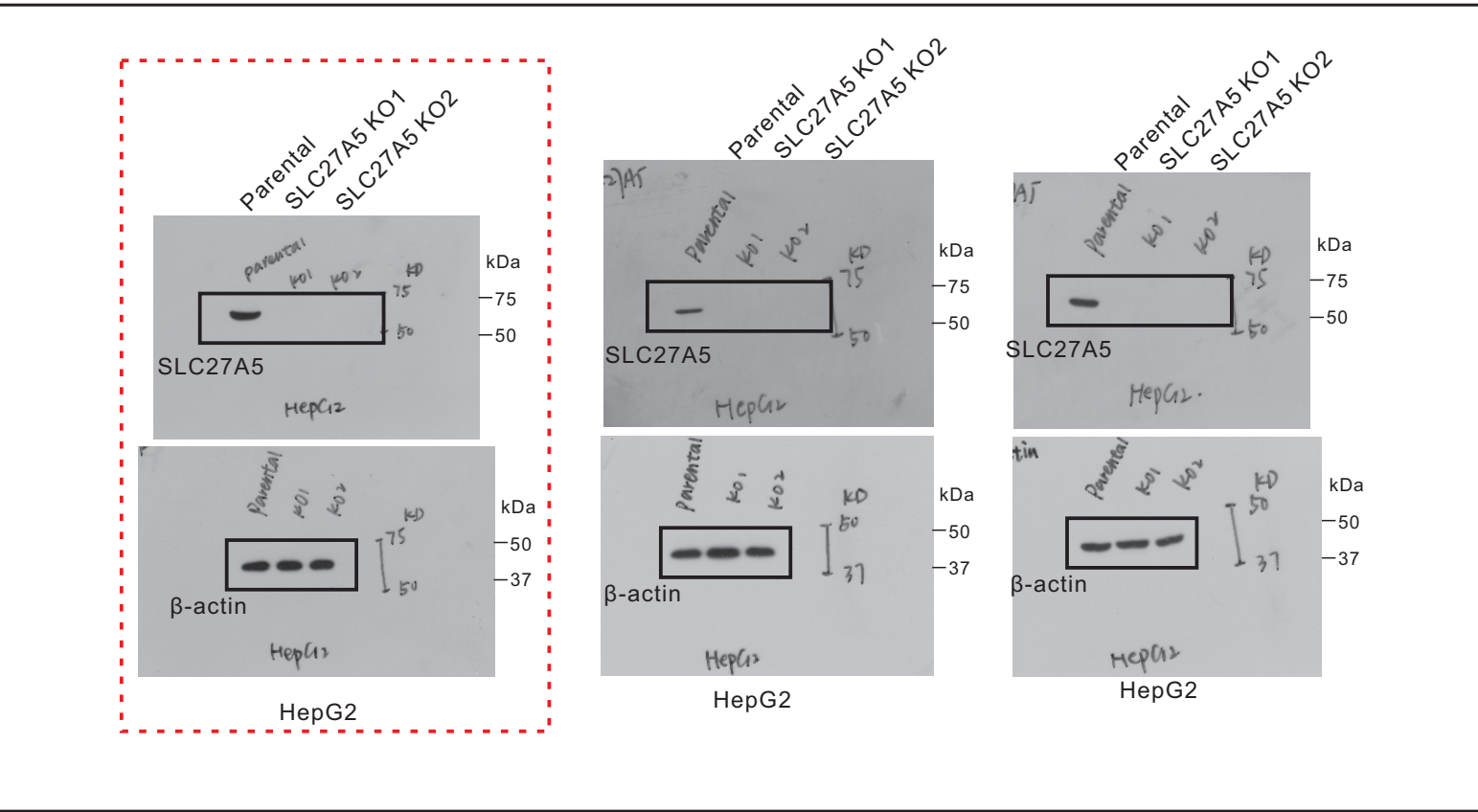

FigS4A

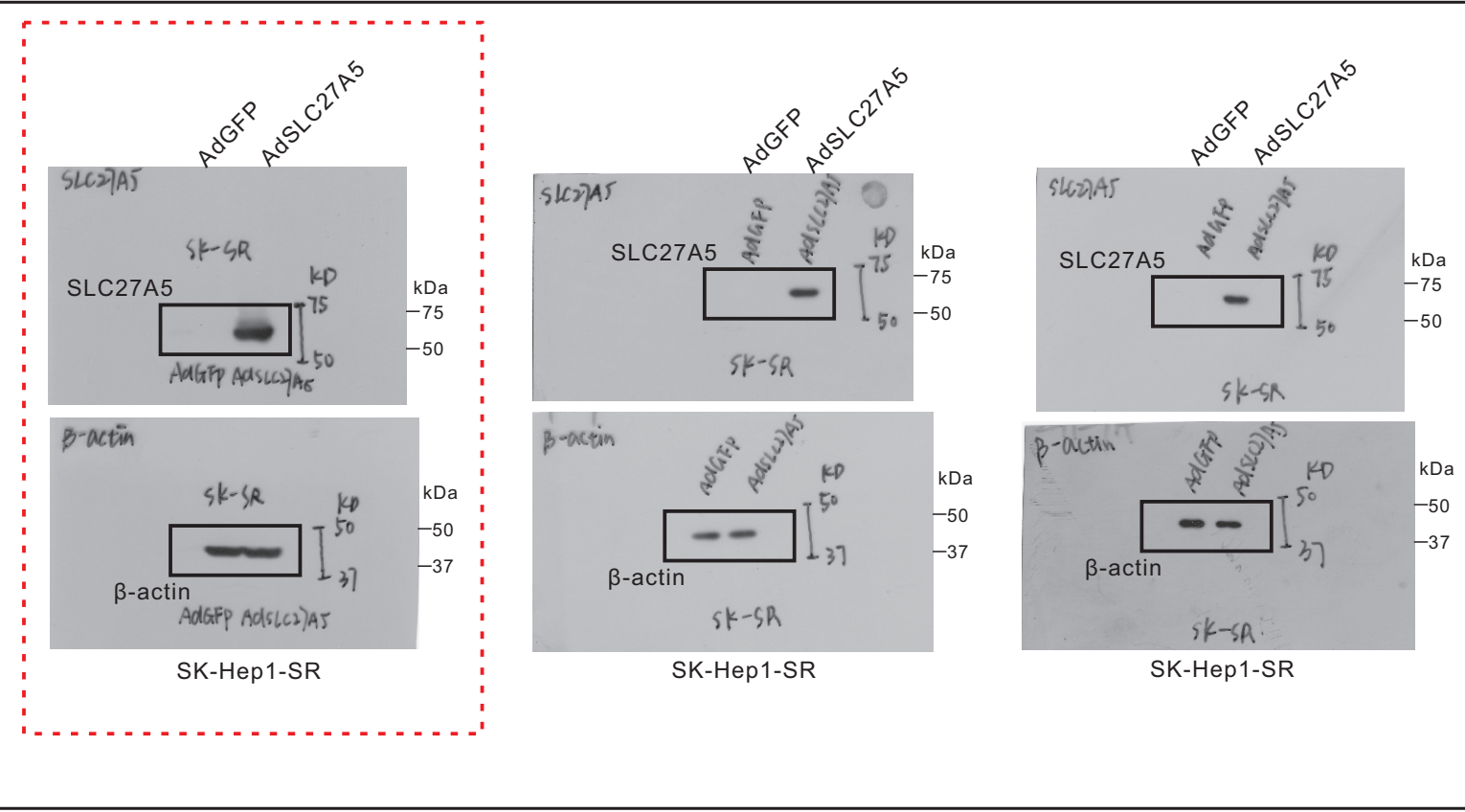

FigS4B

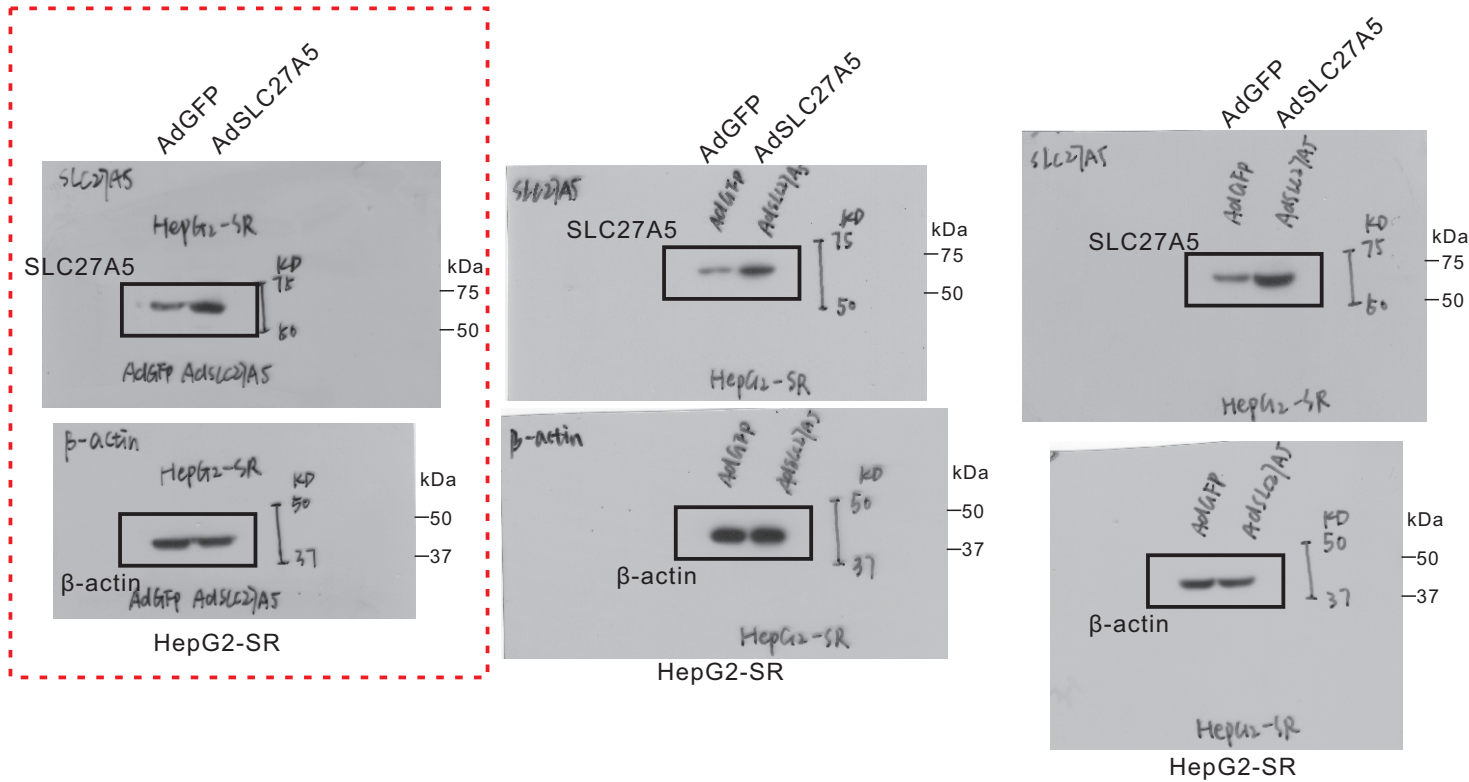

FigS6A

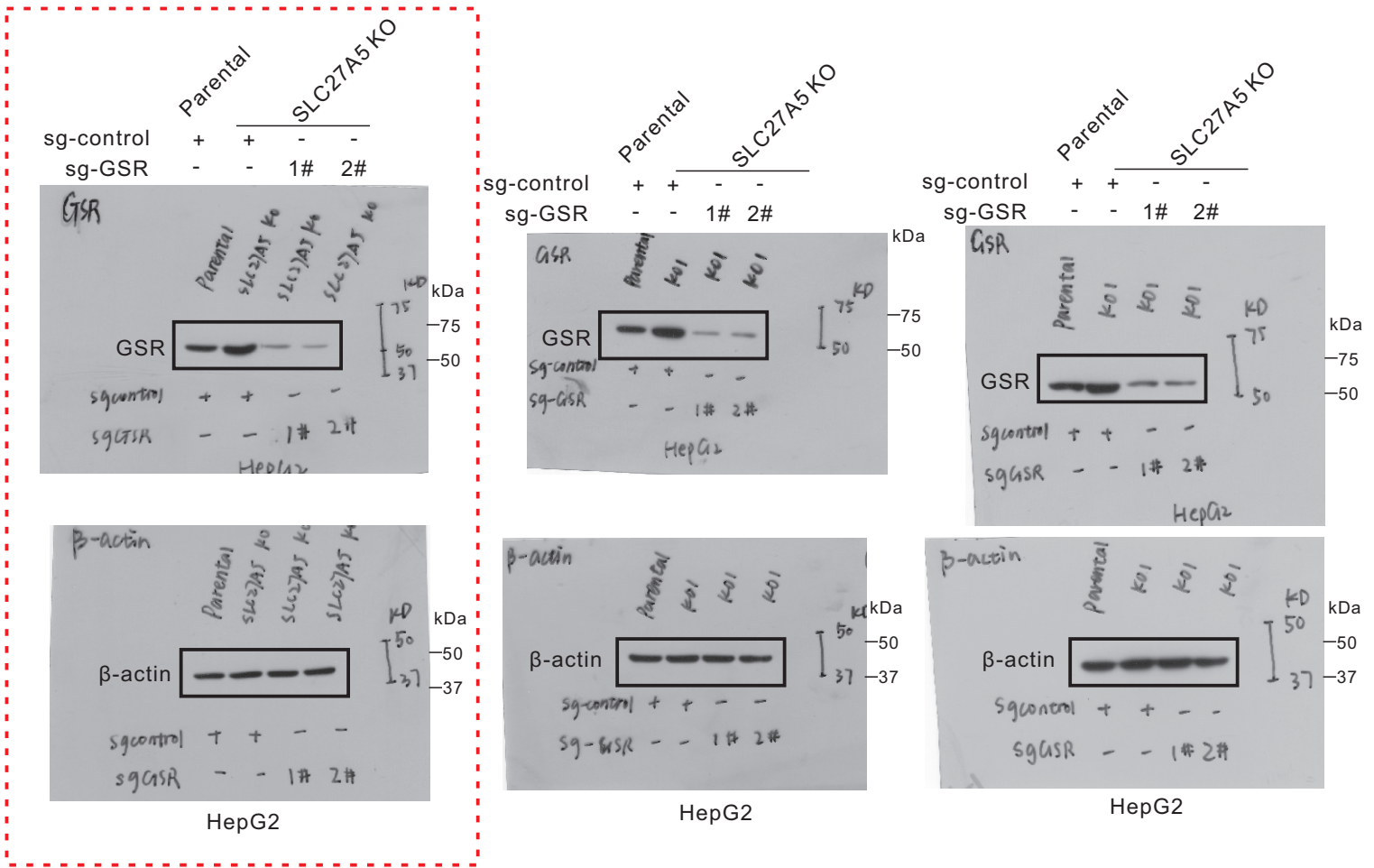

FigS6B

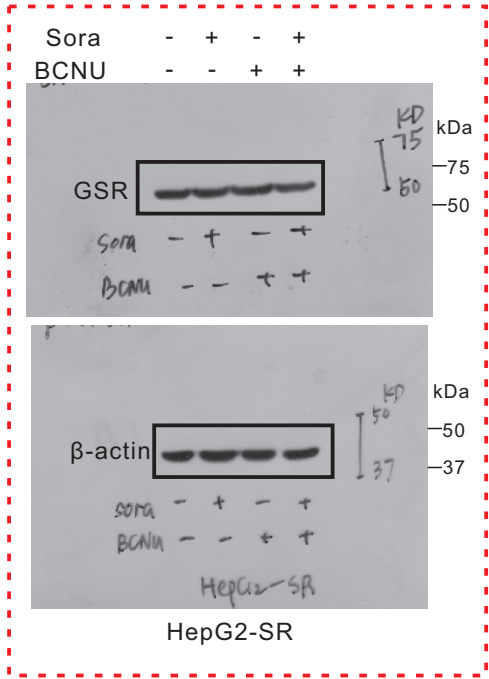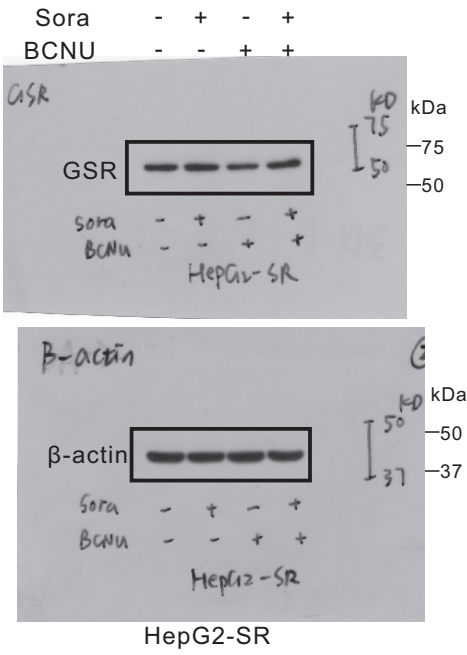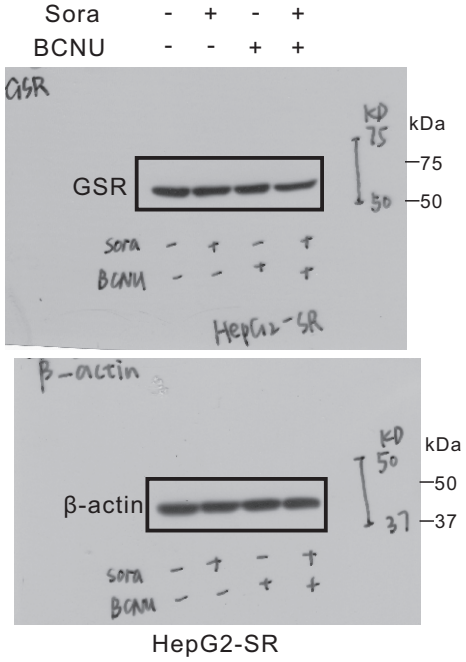

FigS7B

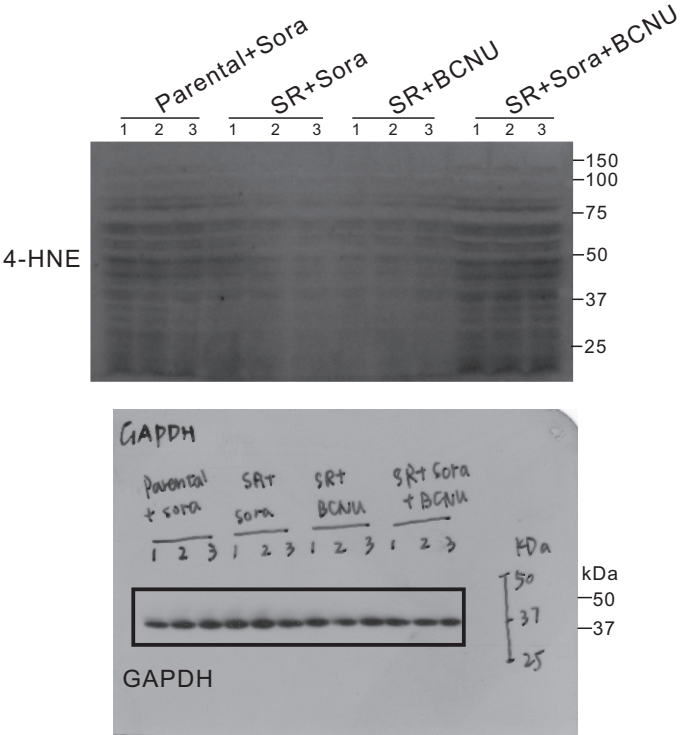

Supplement: Supplementary file 2 — original western blot [file 41419_2023_5558_MOESM2_ESM.pdf]
